# c-FLIP loss links arrest of gene expression to TNFα-dependent antiviral guard immunity

**DOI:** 10.1073/pnas.2600336123

**Published:** 2026-10-22

**Authors:** Matthew Sonnett, Sanne Boersma, Madeline E. Ward, Laura C. Van Eyndhoven, Edoardo Centofanti, Max Brambach, Changhong Zhou, David M. Knipe, Arjun Raj, Alon Oyler-Yaniv, Jennifer Oyler-Yaniv

**Affiliations:** ^a^The Department of Systems Biology, Harvard Medical School, Boston, MA 02115; ^b^ The Department of Bioengineering, School of Engineering and Applied Sciences, University of Pennsylvania, Philadelphia, PA 19104; ^c^Department of Microbiology, Blavatnik Institute, Harvard Medical School, Boston, MA 02115; ^d^ The Department of Genetics, Perelman School of Medicine, University of Pennsylvania, Philadelphia, PA 19104

**Keywords:** antiviral immunity, Herpes Simplex Virus-1, proteomics, mathematical modeling, TNF-alpha

## Abstract

Cells sense infection through dedicated receptors, yet these surveillance pathways are often subverted by pathogen-encoded virulence factors. Guard immunity provides complementary defense by rapidly killing infected cells in response to infection induced stress. However, the symptoms of infection frequently resemble noninfectious stress, raising the question of how they are distinguished. Using quantitative transcriptomics, proteomics, and global translation measurements during viral infections in vitro and in vivo, we show that infection globally suppresses host gene expression and prevents direct antiviral responses. However, the resulting proteostatic stress does not activate guard immunity unless tumor necrosis factor-α (TNFα) is present. We identify the anti-apoptotic protein c-FLIP as a guard for gene expression integrity: its loss sensitizes cells to TNFα, triggering cell death and limiting viral spread. Modeling demonstrates that TNFα and c-FLIP loss function as a biological AND-gate, providing specificity and speed. These findings show how cells integrate internal stress signals with external inflammatory cues to improve the fidelity of death decisions, and how this circuitry may misfire during noninfectious inflammation.

## Significance

Cells suppress gene expression during viral infection, but similar inhibition also occurs during many noninfectious stresses. How cells distinguish these situations has been unclear. Using herpes simplex virus-1 (HSV-1) infection as a model, we show that inhibition of gene expression alone is insufficient to trigger cell death in nonimmune cells. Instead, loss of the short-lived survival protein c-FLIP sensitizes cells to the inflammatory cytokine tumor necrosis factor-α (TNFα), such that the combination of these signals triggers rapid death. This biological AND-gate helps restrict cell death to infected cells while avoiding inappropriate responses to transient noninfectious stress. These findings provide a mechanistic framework for specificity in guard immunity.

The recognition of pathogen associated molecular patterns by dedicated receptors is a central pillar of innate immunity (1). Receptor activation initiates signaling cascades that lead to cytokine production and induction of innate immune gene expression programs. This type of immunity relies on directly sensing components of the pathogen. However, a hallmark of productive infection is the ability of the pathogen to subvert these classical surveillance mechanisms through virulence factors. The ubiquity and diversity of such factors imply that host defenses are constantly undermined during infection, raising the question of how cells and tissues compensate. In addition to direct recognition, cells also rely on indirect sensing of the damage or disruption to the cell caused by the pathogens activity.

This compensatory strategy—known as guard immunity—relies on detecting disruptions to vital cellular processes, such as proteostasis, signaling, or ion flux, thereby linking virulence factor activity to immune activation (2). A prominent example of such disruption occurs when pathogens interfere with host gene expression. Inhibition of transcription or translation has been described during infection by both viruses and certain intracellular bacteria (3, 4). Translational inhibition may arise through virally encoded proteins that interfere with host gene expression, or host stress responses activated during infection. How cells detect these perturbations has emerged as a central question in the study of guard immunity. Recent work shows that the loss of short-lived, pro-survival proteins can serve as a sensitive indicator of pathogen-induced inhibition of host gene expression: Their rapid decay activates cell death signaling that restricts pathogen replication (5, 6). In this framework, the half-life of a protein encodes its potential guard activity: Only fast-decaying proteins can provide a timely readout of disrupted gene expression.

While this mechanism explains how translational shutdown can be detected, it raises the question of how specificity is achieved. Many common and noninfectious stressors, such as nutrient deprivation, ultraviolet radiation, or endoplasmic reticulum stress, also inhibit translation through activation of the integrated stress response (7). It is unclear how a cell distinguishes between transient, noninfectious translational inhibition and pathogen-driven blockade of host gene expression.

One clue to this specificity may lie in the context in which gene expression is perturbed. Similarly to the “Danger Model” in innate immunity, where external cues such as PAMPs, DAMPs, or cytokines are needed to contextualize self-non-self discrimination (8). During infection, gene expression arrest occurs alongside a characteristic cytokine milieu generated by innate immune sentinels. Macrophages, which are widely distributed across host tissues and constitutively express pattern recognition receptors (9), are among the first to sense and respond to pathogens. Upon activation, they release pro-inflammatory cytokines including interleukins (IL)-1, 6, and 12, type I and II interferons, and tumor necrosis factor-α (TNFα).

Specificity alone is insufficient: The death decision must also be faster than the rate of pathogen replication to prevent spread. Our previous work demonstrated that TNFα dramatically hastens cell death in virus-infected cells, limiting viral spread (10). This shows that rapid, context-dependent death decisions can occur, although the molecular mechanisms implementing this combinatorial logic remained unclear.

Here we show that herpes simplex virus-1 (HSV-1) infection causes a global shutdown of host gene expression, primarily through a combination of virus-encoded endonuclease and translational arrest. This shutdown blocks antiviral gene expression, but does not alone trigger rapid cell death. Instead, TNFα provides a contextual cue that licenses rapid death selectively in infected cells, while largely sparing healthy cells. Quantitative multiomics reveals that gene expression arrest drives decay of short-lived proteins, including the anti-apoptotic factor c-FLIP, whose loss acutely sensitizes cells to TNFα. Modeling of the apoptotic network demonstrates that TNFα and c-FLIP decay operate as an AND-gate, producing an autocatalytic death reaction only when both signals coincide. This design confers specificity—restricting death to infected cells—and speed, allowing the response to outpace viral replication and spread. This mechanism distinguishes infectious from noninfectious stress in the activation of mammalian guard immunity.

## Results

### Host Antiviral Genes Are Enriched Following HSV-1 Infection, But mRNA Is Globally Depleted.

**Fig. 1.**
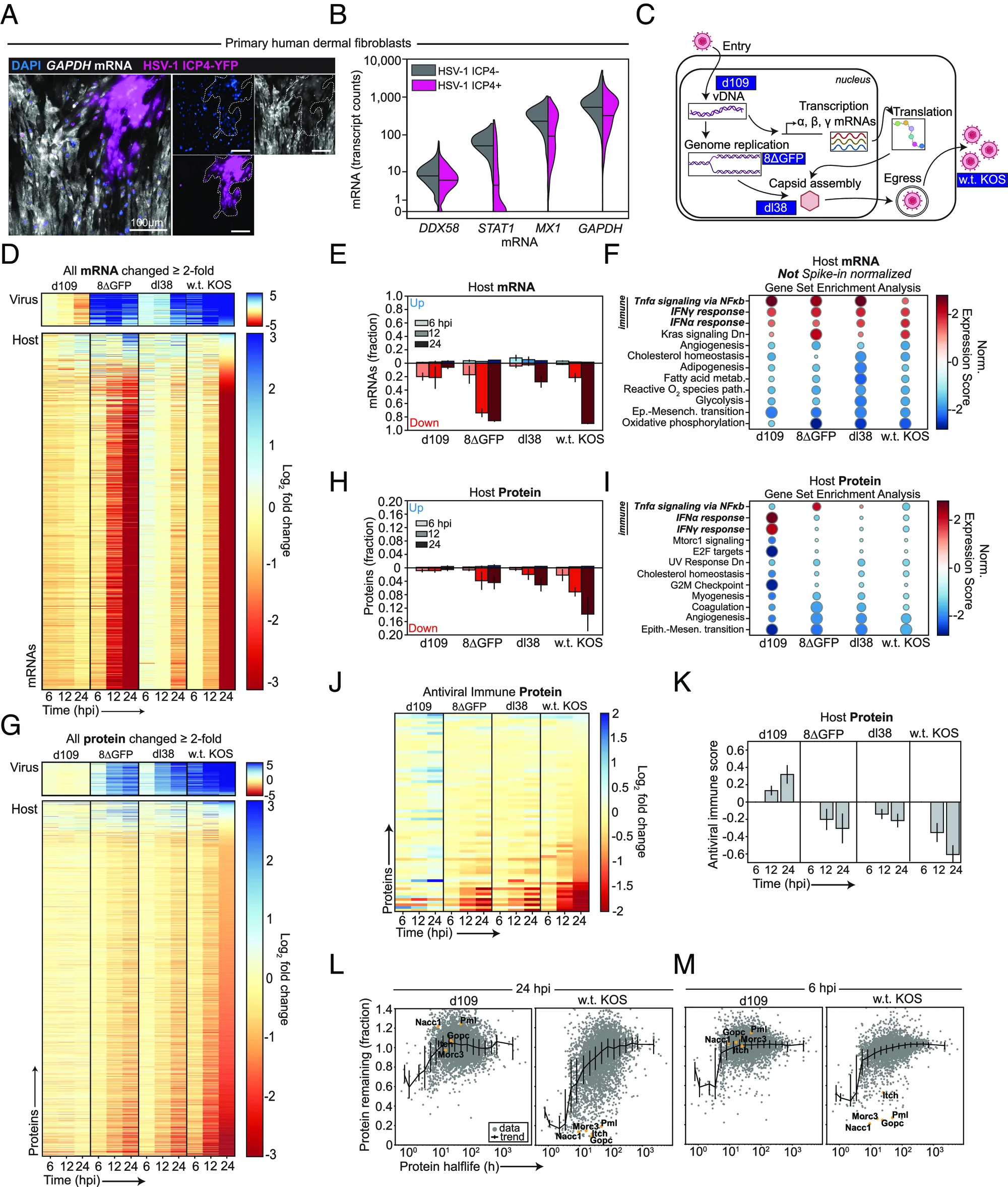
Viral infection leads to genome-wide down-regulation in gene expression and immune impairment. (*A*) Representative FISH image of primary human dermal fibroblasts infected with MOI 0.01 of HSV-1 ICP-YFP (strain 17) and probed for *GAPDH* mRNA. The viral plaque edge is marked with a dotted white line. (*B*) Violin plots quantifying each of the indicated transcripts for human dermal fibroblasts that are either positive or negative for HSV-1 infection (based on ICP4-YFP), aggregated from three technical replicates. (*C*) Diagram showing where each viral mutant is disrupted within the HSV-1 viral life cycle (all on KOS background). (*D*) Heatmap showing all viral and host mRNA that change by ≥twofold relative to the control (uninfected) condition for mouse 3T3 fibroblasts infected with the indicated strains. All transcripts are ranked according to the 24 h time point of the wild type virus-infected cells and represent the mean expression of two biological replicates. (*E*) Quantification of the fraction of host mRNAs that are increased or decreased by ≥twofold relative to the control condition in infected mouse 3T3 fibroblasts. The data are presented as the mean ± SD of two biological replicates. (*F*) Dot plot showing GSEA results for mouse 3T3 fibroblasts infected with the indicated HSV-1 strains at 24 hpi, ranked by log2 fold-change versus mock (CPM-normalized). The top 12 Hallmark pathways by absolute normalized expression score (NES) are shown (FDR < 0.05, |NES| > 1.5). Dot color indicates enrichment direction (NES); dot size indicates significance (−log_10_ FDR). (*G*) Heatmap showing all viral and host proteins that change by ≥twofold relative to the control (uninfected) condition for mouse 3T3 fibroblasts infected with the indicated strains. All proteins are ranked according to the 24 h time point of the wild type virus-infected cells and represent the mean expression of two biological replicates. (*H*) Quantification of the fraction of host proteins that are increased or decreased by ≥twofold relative to the control condition in infected mouse 3T3 fibroblasts. The data are presented as the mean ± SD of two biological replicates. (*I*) Dot plot showing protein abundance GSEA results for mouse 3T3 fibroblasts infected with the indicated HSV-1 strains at 24 hpi, ranked by log2 fold-change versus mock. The top 12 Hallmark pathways by absolute NES are shown (FDR < 0.05, |NES| > 1.5). Dot color indicates enrichment direction (NES); dot size indicates significance (−log_10_ FDR). (*J*) Heatmap showing protein expression for our compiled list of antiviral immune genes for mouse 3T3 fibroblasts. (*K*) Quantification of the antiviral immune score for mouse 3T3 fibroblasts infected with each of the indicated HSV-1 strains. The score represents the mean log2 fold-change of antiviral immune proteins relative to the 6 hpi time point of the same condition ± SEM. (*L* and *M*) Quantification of the fraction of each protein compared to the uninfected control versus its published half-life (15) for infected mouse 3T3 fibroblasts at 24 hpi (*L*) or 6 hpi (*M*). The black trendline shows the average expression ±the 25 and 75% CI for proteins binned by half-life. Known virus-targeted proteins are annotated and shown in orange.

Attenuated viruses vary in the extent to which they interfere with normal cell physiology. We were therefore curious how attenuated viruses reshape the host cell intrinsic immune response. However, we first needed to establish an approach to accurately quantify host transcript abundance during viral infection. HSV-1 infection promotes widespread mRNA depletion via several mechanisms, complicating the interpretation of conventional relative transcript measurements (11–13). We began by visualizing host transcripts directly in primary human dermal fibroblasts infected with wild-type fluorescent-tagged HSV-1 (14), asking if changes in antiviral genes could be distinguished from broader changes in host RNA abundance. We performed fluorescence in situ hybridization, probing for several interferon stimulated genes (ISGs) and *GAPDH*, observing depletion of total RNA within infected cells, although to varying extents for different genes. (Fig. 1 *A* and *B*).

These observations suggest that comparisons based on relative RNA abundance would mask the effects of global host RNA depletion. To account for this, we used RNA-seq normalized with exogenous ERCC spike-in controls (13) to quantify transcript abundance in mouse fibroblasts infected at MOI 10 with a panel of HSV-1 mutants of strain KOS (GEO GSE339037, Dataset S1). This MOI resulted in >90% of the cells becoming infected from the initial viral inoculum (*SI Appendix*, Fig. S1*A*). These mutants complete progressively later stages of the viral replicative cycle: they are defective in activation of viral gene expression [*d*109 (16)], genome replication [8ΔGFP (17)], and capsid assembly (dl38) (Fig. 1*C*). Henceforth, these strains will be referred to as strongly attenuated (*d*109), moderately attenuated (8ΔGFP and dl38), and wild type, and we use the term attenuation to describe impairment of the viral replication cycle. We observed significant and widespread loss of host mRNAs in cells infected with each of the viral strains except the strongly attenuated strain, despite dramatic up-regulation of viral transcripts (Fig. 1 *D* and *E*). When the same data were normalized using counts per million (CPM) rather than ERCC spike-ins, numerous host transcripts appeared to be upregulated, highlighting the importance of spike-in controls for interpreting the results (*SI Appendix*, Fig. S1*B*).

To determine the extent to which host RNA depletion depended specifically on the virion host shutoff protein (vhs), we compared mRNA abundance following infection with the vhs-null mutant UL41NHB and its marker-rescued derivative UL41NHB-R (18). As expected, host mRNA depletion was reduced during infection with UL41NHB relative to UL41NHB-R (*SI Appendix*, Fig. S1*C*). Consistent with previous studies, the contribution of vhs became increasingly apparent at later stages of infection, although appreciable host RNA depletion persisted in its absence, supporting additional vhs-independent mechanisms of host shutoff (11, 13).

ERCC-normalized RNA-seq demonstrated nearly universal depletion of host transcripts, yet gene set enrichment analysis (GSEA) of preranked conventional CPM normalized data at 24 hpi showed that antiviral transcripts became enriched within the remaining transcriptome during infection with each of the viral mutants (Fig. 1*F*) (19). This is consistent with previous work showing that HSV infection does not abolish de novo transcription, but instead establishes a dynamic balance between ongoing RNA synthesis and vhs-mediated degradation (20). Together, these observations raise the possibility that global host RNA depletion shifts the composition of the transcriptome toward newly synthesized antiviral transcripts. We next asked whether this transcriptional remodeling was reflected at the level of protein expression by performing quantitative proteomic analysis of cells infected with the panel of HSV-1 mutants.

### Viral Attenuation-Dependent Depletion of Host Proteins.

We performed 18-plex, quantitative, and time-resolved mass spectrometry-based proteomics on mouse fibroblasts infected with our HSV-1 mutants. We quantified approximately 11.5 thousand total host proteins and 71 viral proteins, or 98% of known HSV-1 proteins (PRIDE #PXD053765; Datasets S2 and S3). Viral proteins were dramatically up-regulated in infected cells, with exception of cells infected with the strongly attenuated strain (Fig. 1*G*). The moderately attenuated strains exhibited reduced viral protein expression relative to the wild type. Of host proteins, between 3 and 20% changed by more than twofold, and most proteins were reduced relative to the uninfected controls (Fig. 1*H*). The degree of protein down-regulation scaled inversely with viral attenuation: more attenuated strains resulted in less protein depletion.

We used the fact that both moderately attenuated strains cannot spread, to test how varying the extent of infection impacts protein depletion. We infected human keratinocytes, a natural target of HSV-1, with different levels of HSV-1 strains dl38 or 8ΔGFP, and performed proteomics at 24 hpi (PRIDE #PXD064114; Dataset S4). Similar to mouse fibroblasts, HSV-1 infection of human keratinocytes also leads to attenuation-dependent protein depletion (*SI Appendix*, Fig. S1*D*). Second, there is an inverse relationship between the amount of cellular viral proteins and the amount of host proteins: More virus leads to more protein depletion (*SI Appendix*, Fig. S1*E*).

Previous studies have demonstrated a similar predominance of down- versus up-regulated proteins during lytic infection with other viruses including vaccinia virus, HIV, Epstein–Barr, and human cytomegalovirus (12, 21–26). These observations, combined with our own, suggest that genome-wide protein depletion may be common to many viral infections. Our data extend these findings by showing that the extent of protein depletion is quantitatively tied to attenuation and viral load.

### Antiviral Proteins Are Only Induced in Cells Infected with a Strongly Attenuated Virus.

To determine whether the proteins that escaped global suppression represented a coordinated host antiviral response, we performed preranked GSEA of host proteins at 24 hpi, ranking all detected proteins by fold-change relative to mock for each strain. While there was no enrichment for immune-related pathways in cells infected with the wild type or moderately attenuated strains, infection with the strongly attenuated strain led to enrichment of antiviral and interferon-response proteins (Fig. 1*I*). We used this list to compile an antiviral expression score (see *Materials and Methods* for details) and observed that, similar to ERCC spike-in normalized mRNA measurements, many antiviral genes are up-regulated only in cells infected with the strongly attenuated strain, yet down-regulated in response to the other strains (Fig. 1 *J* and *K* and *SI Appendix*, Fig. S1 *F* and *G*). We did not observe a similar pattern of up-regulation in two unrelated sets of proteins, yet did observe down-regulation (*SI Appendix*, Fig. S1 *H* and *I*).

To test whether the pattern of up- and down- regulation was specific to antiviral genes, or reflected a global effect, we compared the distribution of protein fold expression changes to 10,000 randomly selected length-matched gene lists. To measure distribution similarity, we calculated the average Kolmogorov–Smirnov statistic for different gene sets. This shows that the pattern of antiviral protein expression in cells infected with any but the strongly attenuated virus is statistically indistinguishable from that of a random gene set (*SI Appendix*, Fig. S1 *J* and *K*). In contrast, the increase in antiviral protein levels in cells infected with the strongly attenuated strain is statistically significant compared to random, indicating virus-specific up-regulation. Corroborating these results, an expression score for an unrelated gene set shows absence of up-regulation, but comparable attenuation-dependent down-regulation (*SI Appendix*, Fig. S1*L*). These results argue that while immune protein up-regulation by cells infected with the strongly attenuated strain is virus-specific, the antiviral down-regulation observed during wild type infection aligns with a model of global protein decay.

The strongly attenuated mutant is similar to defective or abortive infection, as it enters the cell and deposits its genome and tegument proteins, but cannot transcribe viral genes (16). Previous single cell studies demonstrate that only abortively infected cells initiate a type-I IFN or antiviral response (27–30). Our data corroborate this at the bulk protein level, and suggest that only cells that control the virus very early in infection can initiate an innate immune response. Taken together, our data show that productive viral infection not only impairs the induction of antiviral proteins, but also significantly decreases their baseline expression. Although cells infected with the moderately attenuated and wild type viruses transcribe antiviral mRNAs, those mRNAs are not translated.

If the global protein depletion that we observe is due to widespread gene expression arrest, we reasoned that short-lived proteins would be most strongly affected. We plotted the fraction of remaining protein at 24 hpi versus the published half-lives (15) for each protein in the same cell line. We compared between cells infected with the *d*109 mutant, which causes minimal protein depletion, and the wild type strain, where depletion is significant. This analysis supports our hypothesis that decay is due to gene expression arrest, showing that short-lived proteins are reduced more than those with long half lives (Fig. 1*L*).

HSV-1 specifically degrades a subset of host proteins including Gopc, Itch, Morc3, and Pml (21, 25, 31–33). Indeed, each of these proteins was significantly depleted by 6 hpi and remained so until at least 24 hpi, during infection with the wild type, but not the *d*109 mutant. (Fig. 1*M*). Although some proteins are directly degraded by the virus, the broad immune impairment we observe in virus-infected cells arises as a global, indirect consequence of gene expression arrest.

### Infected Cells Reduce Protein Synthesis Commensurate with Viral Attenuation Levels.

**Fig. 2.**
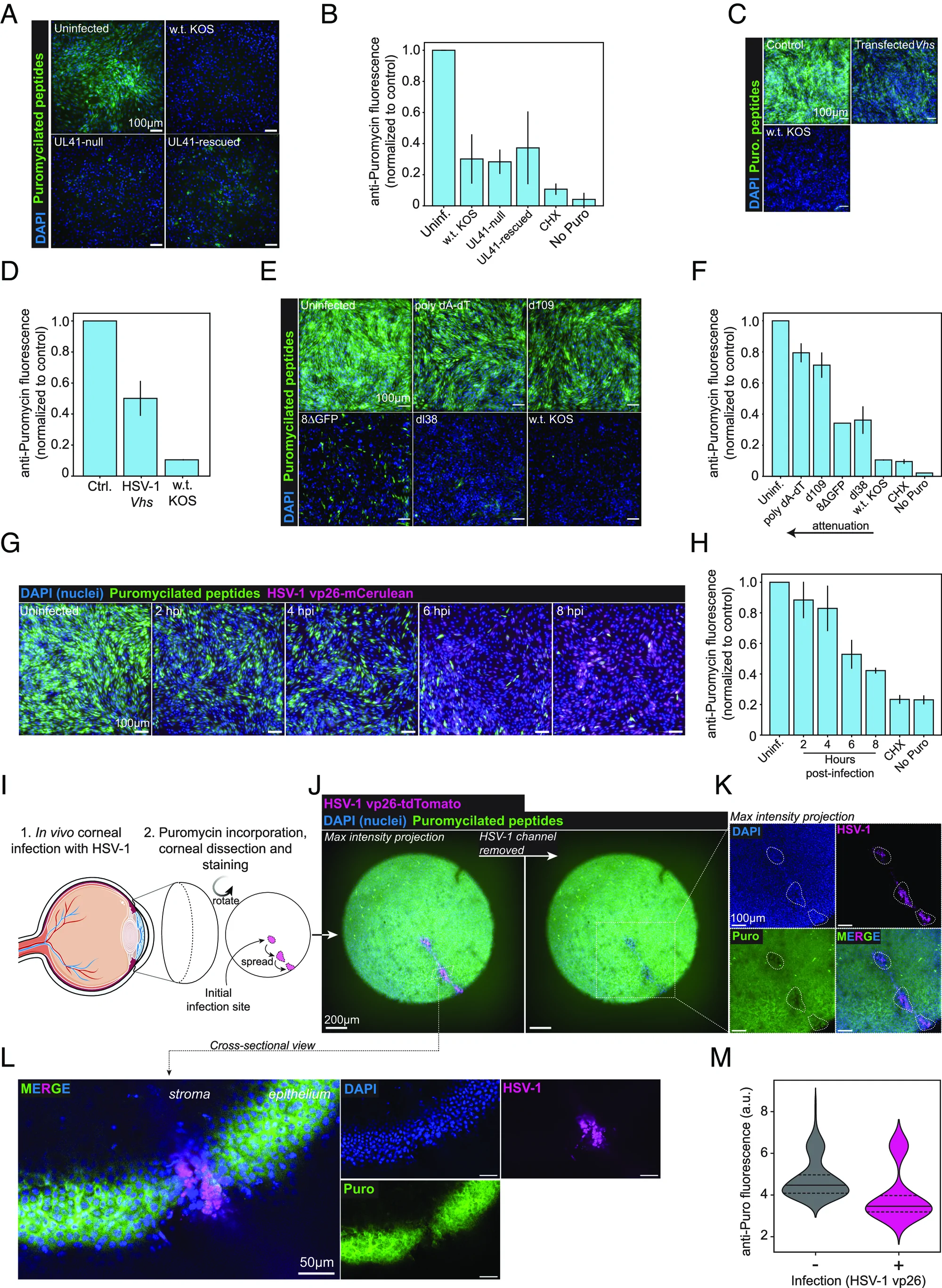
Infected cells reduce protein synthesis commensurate with viral attenuation levels. (*A*) Representative images of puromycin staining for mouse 3T3 fibroblasts infected overnight with MOI 10 of either wild type HSV-1 or vhs null (UL41NHB) or rescued (UL41NHB-R) viruses (all KOS). (*B*) Quantification of average anti-puromycin fluorescence per cell from (*A*) All bar plots show the mean ± SD of multiple biological replicates. (*C*) Representative images of puromycin staining for mouse 3T3 fibroblasts transfected with 200 ng/well of plasmid encoding HSV-1 vhs, or infected with w.t. HSV-1 strain KOS. (*D*) Quantification of average anti-puromycin fluorescence per cell from (*C*). All bar plots show the mean ± SD of multiple biological replicates. (*E*) Representative images of puromycin staining for mouse 3T3 fibroblasts infected overnight with MOI 10 of each of the viral mutant strains (all KOS), or transfected with 200 ng/well of poly dA-dT. (*F*) Quantification of average anti-puromycin fluorescence per cell from (*E*). Bars show the mean ± SD of multiple biological replicates. (*G*) Representative images of puromycin staining for 3T3 fibroblasts infected for varied time with MOI 10 of HSV-1 vp26-mCerulean (KOS). (*H*) Quantification of average anti-puromycin fluorescence per cell from (*G*). Bars show the mean ± SD of multiple biological replicates. (*I*) Cartoon diagram of experimental layout. Mice are infected on debrided corneas with 4 × 10^6^ PFU of HSV-1 vp26-tdTomato. Puromycin incorporation is performed 48 h postinfection, and then whole dissected corneas are imaged using confocal microscopy. (*J*) Representative maximum intensity image of an infected mouse cornea. The virus channel (pink) is removed from the right image to enable visualization of puromycin staining. (*K*) Zoom view of the infection site. (*L*) Close-up view of the indicated cross-section in (*J*). (*M*) Violin plots quantifying anti-puromycin fluorescence for VP26-tdTomato+ (infected) or - (uninfected) cells aggregated from 4 infected animals. Inner lines show the median ± quartiles of the data.

Our data show that HSV-1 infection inhibits host gene expression (Fig. 1). We asked to what extent these observations can be explained by Vhs-mediated mRNA depletion, as HSV-1 also perturbs translation (3). To isolate the global effect of vhs on protein synthesis, we infected cells with wild type, vhs null (UL41NHB), or vhs rescued strains (UL41NHB-R), and quantified active translation by measuring puromycin incorporation into elongating polypeptides (34). The vhs null virus reduced translation to a level comparable to wild type and vhs rescued strain, consistent with prior observations (Fig. 2 *A* and *B*) (13). By contrast, ectopic overexpression of HSV-1 vhs alone reduced, but did not halt protein synthesis (Fig. 2 *C* and *D*), indicating that additional mechanisms suppress translation beyond what would be expected for vhs-mediated mRNA degradation alone. This is consistent with prior observations that many host proteins remain down-regulated during infection with an HSV-1 vhs knockout strain (12, 13). This led us to hypothesize that the global variable protein down-regulation we observed across the different HSV-1 mutant strains (Fig. 1 *G* and *H* and *SI Appendix*, Fig. S1 *D*, *H*, and *I*) is due to suppression in translation.

To test this hypothesis, we quantified protein synthesis after infection with MOI 10 of each HSV-1 mutant strain (Fig. 1*C*). We observed potent reduction in translation commensurate with attenuation in both mouse fibroblasts, and human keratinocytes and epithelial cells (Fig. 2 *E* and *F* and *SI Appendix*, Fig. S2 *A*, *B*, and *G*). We also observed marked reduction in protein synthesis during infection with Influenza A virus (IAV WSN/1933) in both mouse (*SI Appendix*, Fig. S2 *C* and *D*) and human (*SI Appendix*, Fig. S2 *E* and *F*) cells. Translational suppression is restricted only to productively infected cells (*SI Appendix*, Fig. S2 *H* and *I*) (35), and occurs by roughly 8 hpi, consistent with observations from SARS-CoV-2 infection (Fig. 2 *G* and *H*) (36). Taken together, these data demonstrate that infection with variably attenuated HSV-1 strains causes rapid, graded suppression in protein synthesis which are not fully explained by the effects of vhs.

We next tested whether translational suppression occurs during HSV-1 infection in vivo. To do so, we used scarification to infect mice with fluorescent-tagged HSV-1 via a single, central, corneal debridement with a 0.5 mm rotating burr (Fig. 2*I*). The cornea is a natural site of HSV-1 infection in humans, and many aspects of acute infection are well modeled by the murine scarification model (37). Two days postinfection, we observed that protein synthesis was reduced specifically within infected cells (Fig. 2 *J*–*M*). These data demonstrate that HSV-1 infection leads to profound suppression in host gene expression in vivo. More broadly, suppression of translation appears common as it occurs during infection with IAV and in a variety of different mouse and human cells.

### c-FLIP Loss Mediates Sensitivity of Virus-Infected Cells to TNFα, Restricting Viral Spread.

We have shown that infection with HSV-1 rapidly and profoundly inhibits host gene expression, impeding the antiviral response. This raises the question of how translationally repressed cells can confine viral spread. We previously demonstrated that viral infection sensitizes cells to macrophage-derived tumor necrosis factor-α (TNFα), which restricts viral spread (*SI Appendix*, Fig. S3*A*) (10). However, the molecular mechanism responsible for this TNFα sensitivity was unknown. A body of work has shown that translational inhibition leads to the decay of short-lived pro-survival proteins, which sensitizes cells to cytotoxic ligands such as TRAIL or TNFα (38–41). This work, coupled with our observations that HSV-1 halts translation, led us to hypothesize that decay in short-lived pro-survival proteins mediates enhanced sensitivity to TNFα during infection.

To test this hypothesis, we first checked whether the protective effect of TNFα is directly attributable to cell death. We treated cells with a low MOI (1) of fluorescent-tagged HSV-1 (42), with and without a low dose of TNFα (5 ng/mL), and measured viral spread via microscopy. Consistent with our published work, TNFα restricts viral spread, resulting in only small patches of infection compared to untreated cells (*SI Appendix*, Fig. S3 *B*–*F*). Inhibiting programmed death signaling with inhibitors of caspases and RIP kinase (ZVad and Nec1) completely abrogates the protective effect of TNFα. Microscopy images show a high density of dead cells within infection foci in TNFα-treated cultures, that are reduced in the presence of ZVad and Nec1 (*SI Appendix*, Fig. S3 *G*–*I*). We also verified that application of these inhibitors reduces cell death (*SI Appendix*, Fig. S3 *J* and *K*). These data reproduce our previous findings, and extend them by demonstrating that the protective effect of TNFα relies on cell death.

**Fig. 3.**
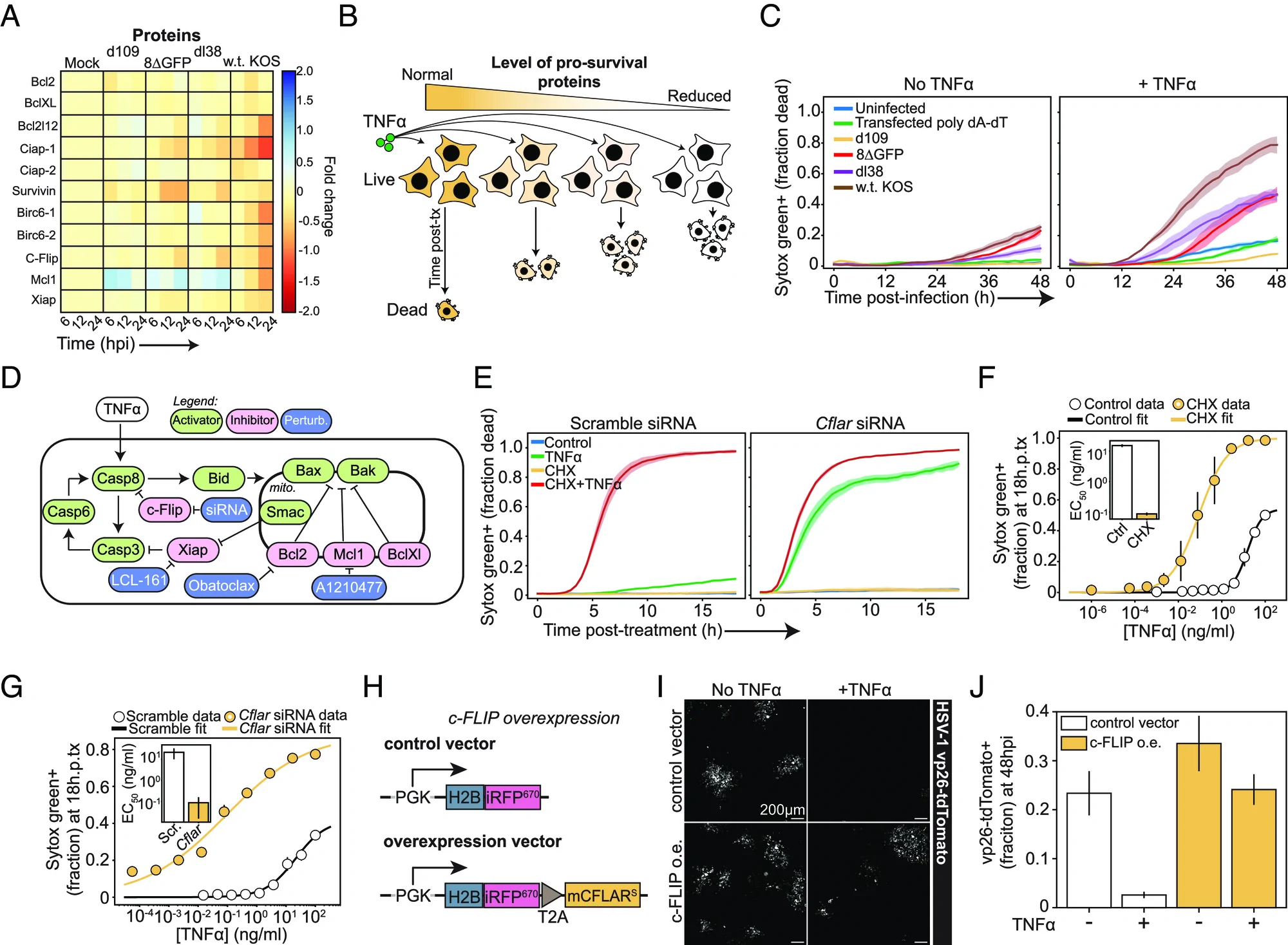
c-FLIP loss mediates sensitivity of virus-infected cells to TNFα, restricting viral spread. (*A*) Heatmap showing protein expression (from proteomics experiment) for our compiled list of pro-survival genes for mouse 3T3 fibroblasts. The data represent the mean expression of two biological replicates. (*B*) Cartoon diagram showing the working model: The expression level of pro-survival proteins tunes the cellular sensitivity to TNFα. (*C*) Quantification of cell death over time for 3T3 fibroblasts infected with MOI 10 of each viral mutant (KOS), or transfected with 200 ng/well of poly dA-dT with or without concurrent treatment with 5 ng/mL TNFα. (*D*) Cartoon diagram of the key positive and negative regulators of the extrinsic apoptotic signaling network, and various experimental perturbation strategies. (*E*) Quantification of cell death over time for 3T3 cells transfected with control or *cflar* siRNAs and the indicated conditions: 20 μM CHX or 5 ng/mL TNFα, or CHX with TNFα. (*F*) Quantification of cell death at 18 hp-tx for 3T3 cells treated with a dose titration of TNFα with or without concurrent treatment with 20 μM CHX. Curves were fit with a Hill function. Error bars show the mean ± SD of technical duplicates. *Inset* shows the TNFα EC_50_ values computed from the fit. Error bars show the SE of the fit. (*G*) Quantification of dead cells for 3T3 cells transfected with either control or *cflar* siRNAs at 18hp-tx with a dose titration of TNFα. Each data point shows the mean ± SD of technical duplicates. Inset shows the TNFα EC_50_ values computed from the fit. Error bars show the SE of the fit. (*H*) Diagram of the constructs used in c-FLIP overexpression experiments. We overexpressed the mouse Raji c-FLIP isoform (Cflar^R^) in 3T3 cells. (*I*) Representative images of viral spread 48 h after MOI 1 infection with HSV-1 vp26-tdTomato for 3T3 cells stably transduced with the control vector, or c-FLIP overexpression vector. (*J*) Quantification of infected cells for each of the indicated conditions at 48 hpi. For Fig. 3, unless otherwise stated, the error bars show the mean ± SD of multiple biological replicates.

We next returned to our proteomics dataset to investigate the effect of viral infection on the expression of a cohort of pro-survival proteins expressed by mouse fibroblasts. Similar to the proteome-wide trend of protein loss, we observed progressive, attenuation-dependent decay of most of the proteins in this network (Fig. 3*A*). We surmised that attenuation-dependent loss of pro-survival proteins would translate into graded sensitivity to TNFα (Fig. 3*B*). To test this, we infected mouse fibroblasts with MOI 10 of each of the different HSV-1 strains (Fig. 1*C*), and transfected poly dA-dT, a mimetic of double-stranded DNA viruses, and visualized cell death by microscopy. Consistent with our past work (10), none of the virus strains was very cytotoxic alone, yet combination with a low dose of TNFα synergistically enhanced cell death in a manner that corresponded with attenuation level (Fig. 3*C*).

Several of the pro-survival proteins in our list (Fig. 3*A*) could mediate sensitivity to TNFα during infection. To identify which might play this role, we targeted them specifically with inhibitors and cotreated with low-dose TNFα (Fig. 3*D*). We anticipated that a hit would phenocopy the effect of cycloheximide (CHX)—an inhibitor of protein synthesis—and TNFα combined (*SI Appendix*, Fig. S3*L*). While inhibition of Bcl2 family proteins, Mcl1, and Xiap did not strongly synergize with TNFα to induce death, transfection of siRNAs targeting *Cflar* (c-FLIP)—which reduce the expression of both the long and shorter Raji isoforms of the protein—phenocopied the dynamics and extent of cell death induced by CHX combined with TNFα alone (Fig. 3*E* and *SI Appendix*, Fig. S3 *M*–*O*). To measure and compare the increase in sensitivity to TNFα conferred by CHX treatment and c-Flip knockdown, we performed dose responses, quantifying cell death at 18 h. At baseline, mouse 3T3 fibroblasts have an EC_50_ of approximately 10 ng/mL TNFα (Fig. 3*F*). Treatment with CHX and c-FLIP knockdown both result in a dramatic and comparable increase in sensitivity of nearly 100-fold to about 0.1 ng/mL (Fig. 3 *F* and *G*). Combined, these data demonstrate that c-FLIP tunes cellular sensitivity to TNFα.

If our hypothesis is correct, we expect c-FLIP overexpression to desensitize cells to TNFα and enhance viral spread. We constructed stable fibroblast lines that overexpress the mouse c-FLIP-R isoform, which has been widely shown to antagonize caspase-8–mediated cell death (43) (Fig. 3*H*). Overexpression of c-FLIP-R eliminated the cells’ sensitivity to TNFα (*SI Appendix*, Fig. S3*P*). Next, we infected control-transduced or c-FLIP-R overexpression cells with a low MOI (1) of fluorescent-tagged HSV-1. In absence of TNFα, both cell lines exhibited similar levels of infection at 48 hpi (Fig. 3 *I* and *J*), yet low-dose TNFα reduced spread only in control-vector transduced cells. This shows that c-FLIP mediates the cells’ sensitivity to TNFα during viral infection, serving an antiviral function. These findings suggest that decay of c-FLIP during HSV-1-driven translational shutdown lowers the threshold for TNFα-mediated death, thereby restricting viral spread.

### A Noise Induced Stochastic Switch Drives the Timing of Death Decisions.

**Fig. 4.**
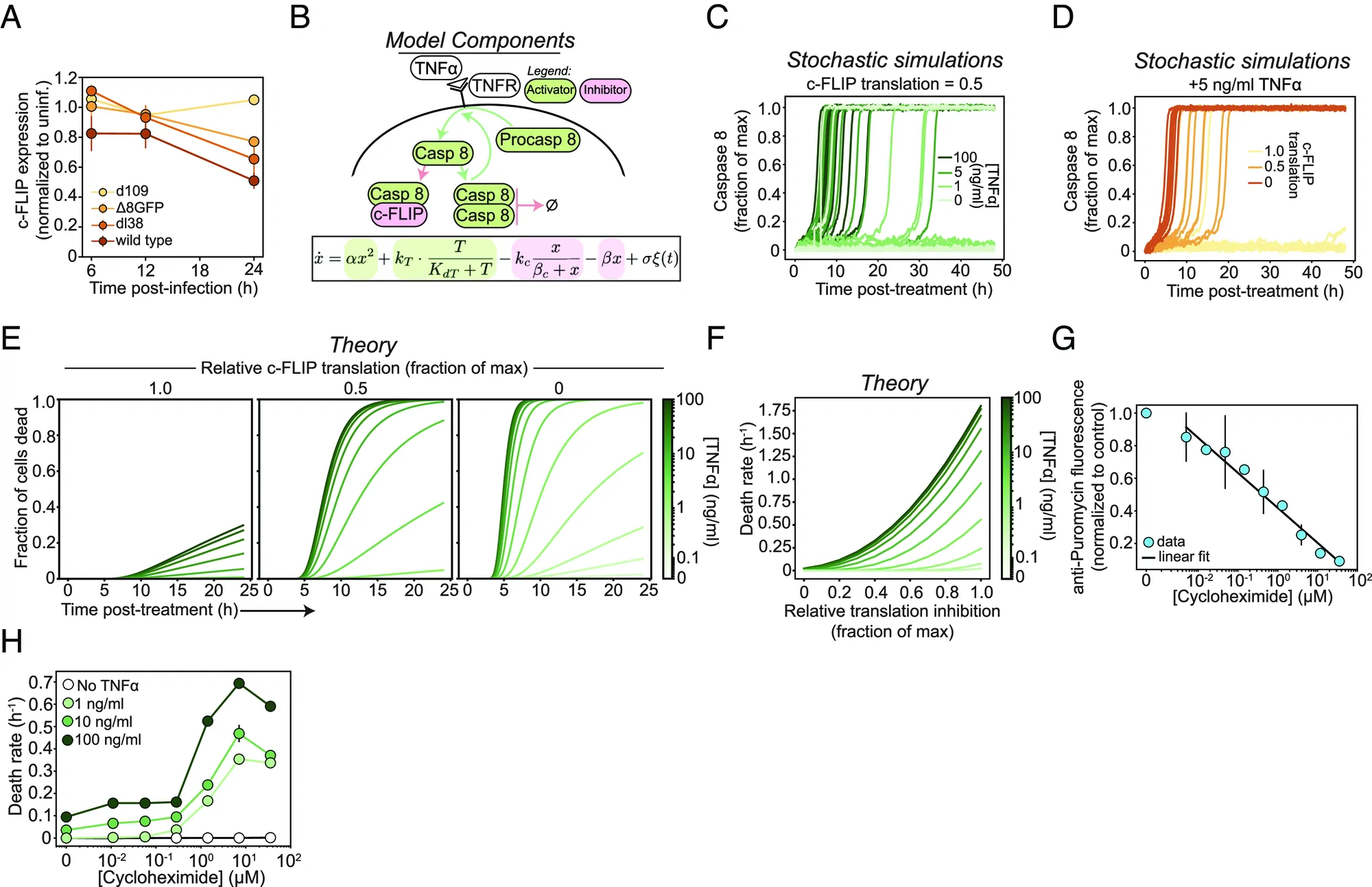
A noise induced stochastic switch drives the timing of death decisions. (*A*) Quantification of c-FLIP over time from proteomics experiments for 3T3 cells infected with each of the viral mutants. Data show the mean ± SD of biological replicates normalized to the control (uninfected condition) at each time point. (*B*) Cartoon diagram of the mathematical model. Our model incorporates TNFα triggered procaspase-8 cleavage, autocatalysis of procaspase-8 by caspase-8 dimers, inhibition of caspase-8 by free c-FLIP, natural caspase-8 degradation, and stochastic biochemical noise. (*C*) Individual trajectories for stochastic simulations of caspase 8 cleavage for four different TNFα concentrations and a c-FLIP translation rate of 0.5 of the maximum. (*D*) Individual trajectories for stochastic simulations of caspase 8 cleavage for three different c-FLIP translation rates and a fixed low concentration of TNFα. (*E*) Model predictions for the proportion of cells killed by treatment with different concentrations of TNFα and three different rates for c-FLIP translation. (*F*) Theory results for the population death rates for a spectrum of different TNFα concentrations and c-FLIP translation rates. (*G*) Quantification of average anti-puromycin fluorescence per cell for 3T3 cells treated with a dose-titration of cycloheximide. Black line shows a linear fit, demonstrating a linear reduction in protein synthesis. (*H*) Quantification of the death rates for 3T3 cells treated with a dose titration of cycloheximide and the indicated concentrations of TNFα. Death rates were fit by converting cell death to log space and linearly fitting the exponential component, as we did previously (10). For all of Fig. 4, unless otherwise stated, data show the mean ± the SD of multiple biological replicates.

We were intrigued by the extent to which even modest decreases in c-FLIP expression synergize with TNFα to induce cell death (Fig. 4*A*). We used mathematical modeling of caspase-8 activation to probe the characteristics of the apoptotic signaling network that permit such dynamics, building on prior quantitative and structural analyses of DISC signaling and caspase-8 regulation (43, 44). Here, we focus on a reduced model capturing the competitive interaction between c-FLIP and caspase-8 and its impact on threshold behavior in cell death.

Signaling in response to TNFα scales with the dose (45, 46), yet there is high variability both between cells and for a given cell over time. Variability in signaling and cell death is driven by differences in protein expression and stochastic biochemical noise (38, 41, 47), necessitating a stochastic differential equation model (Fig. 4*B*). Downstream of the TNFα receptor, procaspase-8 is cleaved into active caspase-8 proportional to receptor activation (39). Activated caspase-8 dimerizes, then cleaves procaspase-8 in an autocatalytic loop (48). c-FLIP acts as a classic competitive inhibitor by binding to caspase-8, preventing dimerization (41, 43, 49). In addition, caspase-8 undergoes proteasomal degradation (41, 50).

Conceptually, the rate of c-FLIP translation dictates the cells’ ability to counteract active caspase-8. If caspase-8 levels saturate available c-FLIP, positive feedback will dominate and the cell will die. Biochemical noise plays a crucial role as it can tip the levels of caspase-8 above the saturation limit, triggering death (38, 41, 47). TNFα increases the baseline level of caspase-8, such that a smaller—and therefore more likely—fluctuation will cause death (Fig. 4*C*). Reduced c-FLIP translation lowers the threshold for caspase-8 positive feedback activation, resulting in faster and less variable timing of cell death (Fig. 4*D*).

After an initial lag caused by transition of signaling away from homeostasis, which we observe experimentally (Fig. 3*E* and *SI Appendix*, Fig. S3*L*), different levels of TNFα and c-FLIP translation cause an exponential increase in death (Fig. 4*E*). Our model predicts that in the presence of TNFα, a linear decrease in protein translation will cause a superlinear increase in cell death (Fig. 4*F*). Alone, low to moderate concentrations of TNFα or c-FLIP reduction are insufficient to cause much death. However, TNFα synergizes with decreases in c-FLIP translation, driving rapid cell death. To test these model predictions, we treated cells with a dose-titration of CHX, causing a linear decrease in protein synthesis (Fig. 4*G*) and measured death rates across different concentrations of TNFα. As predicted, we observed a superlinear relationship between the degree of translation inhibition and cell death (Fig. 4*H*). Even a modest twofold drop in c-FLIP was sufficient to accelerate cell death by orders of magnitude.

Our model suggests, and experiments reinforce, an elegant molecular logic whereby c-FLIP levels regulate the probability that caspase-8 positive feedback, and resulting cell death, will be triggered in response to TNFα. This mechanism enables a remarkably wide range of death rates to be achieved depending on the extent of translational suppression and TNFα. These findings show that virus-driven cessation of protein synthesis leads to rapid decay of the short-lived survival protein c-FLIP, lowering the threshold for TNFα-mediated cell death.

### Cells Subject to Noninfectious Sources of Stress Die When Exposed to TNF.

Many noninfectious stressors transiently inhibit translation, but pose no broader threat to the organism. However, the aberrant release of TNFα has deleterious effects in many disease contexts. Our data suggest that the release of TNFα in contexts of noninfectious translational stress may inappropriately trigger guard immunity and cell death, despite the absence of a pathogen. To test this hypothesis, we exposed cells to several stressors either alone, or in combination with TNFα, and quantified death over time.

**Fig. 5.**
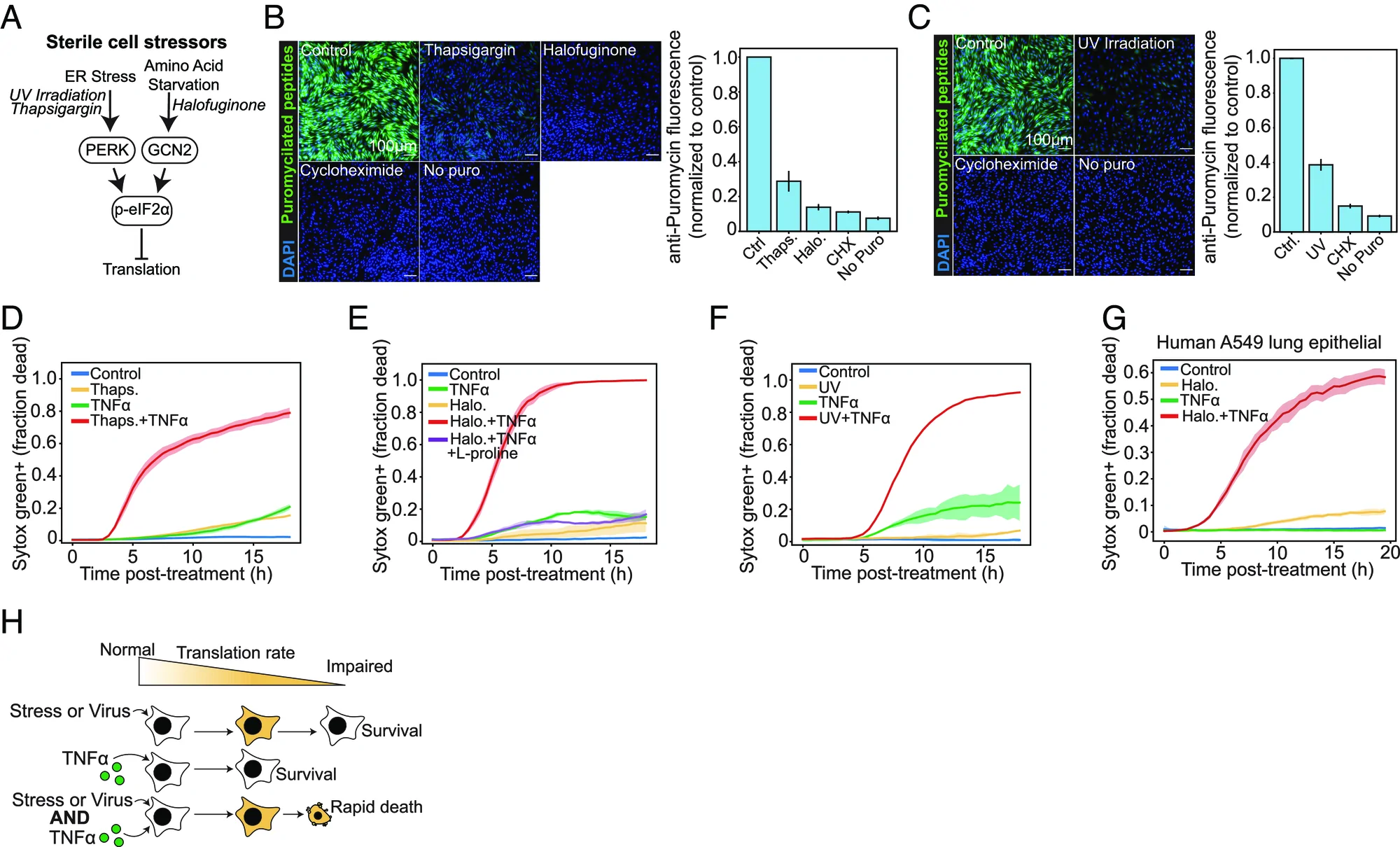
Cells subject to noninfectious sources of stress die when exposed to TNF. (*A*) Cartoon diagram showing examples of stressors that activate the ISR and drug inducers we use in our experiments. (*B*) Representative images of anti-puromycin staining for 3T3 cells treated with either 1 μM Thapsigargin or 500 nM Halofuginone for 6 h prior to puromycin pulse, along with quantification of average anti-puromycin fluorescence per cell for each of the different conditions. (*C*) Representative images of anti-puromycin staining for 3T3 cells that were UV irradiated with 150 J/m^2^, along with quantification of average anti-puromycin fluorescence per cell for each of the different conditions. (*D*) Quantification of cell death over time for 3T3 cells treated with 5 ng/mL TNFα or 1 μM Thapsigargin, or TNFα and Thapsigargin. (*E*) Quantification of cell death over time for 3T3 cells treated with 5 ng/mL TNFα or 500 nM Halofuginone, or TNFα and Halofuginone, or TNFα and Halofuginone and 4 mM L-Proline. Proline competes for the target of Halofuginone (prolyl tRNA synthetase). Hence, proline elimination of cytotoxicity indicates that cytotoxicity arises from Halofuginone’s on-target mechanism. (*F*) Quantification of cell death over time for 3T3 cells treated with 5 ng/mL TNFα or 150 J/m^2^ UV irradiation, or TNFα and UV irradiation. (*G*) Quantification of cell death over time for A549 cells treated with 5 ng/mL TNFα or 500 nM Halofuginone, or TNFα and Halofuginone. (*H*) Cartoon depicting the working model. Both noninfectious stressors and viral infection can cause translational impairment in cells. However, in these cases, cells do not readily die. Low doses of TNFα also do not cause significant cell death. Combining translational inhibition due to stressors or viral infection causes rapid cell death. For all of Fig. 5, all of the data show the mean ± SD of multiple biological replicates.

The integrated stress response (ISR) responds to a broad array of different stressors by triggering one of four kinases that phosphorylate the downstream eIF2α, halting translation of most mRNAs (7). To activate the ISR, we used UV irradiation and Thapsigargin, which induce ER stress and the kinase PERK; and Halofuginone, which stimulates the amino acid starvation response and the kinase GCN2 (Fig. 5*A*) (7, 51). Consistent with the established consequences of each treatment, we observed significant inhibition of protein synthesis (Fig. 5 *B* and *C*). Although none of the treatments were cytotoxic alone, cotreatment with a low dose of TNFα strongly synergized to induce cell death in mouse fibroblasts and human lung epithelial cells, similar to viral infection or translation inhibitors (Fig. 5 *D*–*G*). These data indicate that synergy in cell death induced by translational inhibition and TNFα is a general mechanism, because it is independent of the cause for translational arrest (Fig. 5*H*). Because TNFα is released by activated macrophages during infection, we argue that it contextualizes the loss of translation as infectious, thereby licensing guard immunity in inflamed tissues. Conditioning the synergy in cell death on an immune-derived cue safeguards cell viability during noninfectious disruptions to protein synthesis.

## Discussion

In this study we asked how specificity is achieved in the decision to trigger guard immunity following HSV-1 infection. We show that infection impedes host gene expression via both transcription and translation, with moderately attenuated and wild type viruses causing stronger arrest. Consequently, productively infected cells fail to trigger a canonical antiviral protein response. Although translational inhibition can also arise as part of innate immune stress responses, including ISR activation downstream of viral replication (52), our data indicate that the pronounced shutdown observed during HSV infection depends on productive infection rather than direct sensing of viral nucleic acids alone. Thus, irrespective of the precise upstream mechanism, the relevant signal reflects disruption of host translation during productive infection, consistent with a guard immunity framework (2).

Previous studies have pointed to decay in pro-survival proteins as a mechanism to survey for disruptions to protein synthesis (5, 6), but how cells distinguish infectious from noninfectious translational stress was unclear. We find that translational suppression alone is not sufficient to trigger cell death in HSV-1-infected cells. Contextual cues such as TNFα from neighboring immune cells license rapid death selectively in infected cells (10). Translational shutdown during HSV-1 infection drives decay of the short-lived survival protein c-FLIP, sensitizing infected cells to TNFα-dependent death and limiting viral spread.

### Productive Infection Impedes Cellular Activation of an Antiviral Response.

Although the interferon response is central to antiviral immunity, our data show that viral replication is mutually incompatible with host translation of interferons or ISGs (1, 36, 53, 54). Consequently, infection with a highly attenuated virus induces a canonical antiviral protein response, whereas infection with moderately attenuated or wild type strains does not. The failure to upregulate immune proteins implies that productively infected cells must rely on alternative forms of defense. We suggest that, in vivo, only cells that are abortively infected or uninfected cells neighboring them, will up-regulate immune proteins (27).

### Viral Infection Causes Progressive and Attenuation-Dependent Translational Inhibition.

We show that the inhibition of antiviral defense is not pathway specific, but rather a consequence of a global shutdown that proceeds via both mRNA depletion and translational inhibition. This global shutdown cannot be explained solely by the effects of HSV-1’s endonuclease vhs, implying a contribution from additional factors derived from either virus, host, or both. The mechanism responsible for the nearly complete translational inhibition observed during infection with the wild type strain remains to be determined. The degree of translational inhibition scales inversely with attenuation, with wild type virus causing a complete cessation in gene expression.

### c-FLIP Acts as a Context-Dependent Guard Protein During Viral Infection.

The impact of translational inhibition is particularly severe for fast-degrading proteins, which decay quickly after global translational inhibition. Mechanistically, we showed that this drives decay of the short-lived anti-apoptotic protein c-FLIP. c-FLIP exists in multiple isoforms, including the long (c-FLIP-L) and shorter Raji (c-FLIP-R) variants that differentially regulate caspase-8 signaling at the death-inducing signaling complex (43, 55). siRNA reduces total c-FLIP expression, whereas our gain-of-function experiments restore the Raji isoform (c-FLIP-R), which is well established to antagonize caspase-8–mediated cell death.

The decay of c-FLIP potentiates an ultrasensitive switch that is triggered in response to TNFα, rapidly killing the infected cell. Mathematical modeling provides clarity into the molecular logic that enables synergy between c-FLIP decay and death receptor signaling: Through its’ action as a competitive inhibitor of caspase-8 autocatalysis, c-FLIP modulates a stochastic molecular switch controlling induction of caspase-8 positive feedback and triggering of cell death. Even a modest reduction in c-FLIP dramatically sensitizes cells to TNFα-induced caspase-8 activation, making cells die faster. The stochastic noise present in the TNFα signaling network is instrumental for this process, conferring an antiviral function.

### Viral Attenuation Modulates Sensitivity to TNFα.

To serve as an effective antiviral strategy, cells must die before the completion of the viral life cycle. This could imply that very rapid death is always preferable, yet, cell death itself poses risks to the organism. We showed that cellular sensitivity to TNFα is quantitatively tied to attenuation. By linking the rate of cell death to the extent of viral attenuation, cells are able to preserve tissue integrity and function for longer, while also preventing viral spread (10). This suggests potential optimality in the cellular circuits that evolved to serve a physiological role, in this case preserving barrier function, while also defending against invading pathogens.

### AND-Gate Logic Improves Fidelity for Costly Cell Death Decisions.

The decision to undergo cell death is intrinsically costly, requiring the host to expend resources to restore tissue integrity and maintain function. Although perturbations in gene expression are a hallmark of productive viral infection, similar disruptions can arise from noninfectious environmental stressors. Likewise, TNFα is released by activated innate immune cells during infection and can act broadly on nearby cells, independent of their infection status. By conditioning cell death on the simultaneous presence of both translation inhibition and TNFα, an effective logical AND-gate, the host improves specificity by reducing spurious cell loss that would occur if either signal acted alone.

### Erroneous or Incidental TNFα Exposure May Jeopardize Otherwise Viable Cells.

Our study suggests that even in the absence of infection, the aberrant production of TNF superfamily cytokines may have deleterious effects on translationally stressed cells. This is often the case during later stages of viral disease, where the infection itself has been resolved, but the surrounding tissue still suffers from stress (56). Indeed, rapid and widespread cell death is characteristic of such clinical conditions, and blocking TNFα signaling has been shown to improve barrier and tissue function (57). Future work should examine the role of translational inhibition in cell death during barrier regeneration during postviral syndromes, and other noninfectious stress settings.

## Materials and Methods

### Animals.

All experiments were performed according to the Guide for the Care and Use of Laboratory Animals and were approved by the Institutional Animal Care and Use Committee at Harvard Medical School. C57Bl6/J mice were purchased from the Jackson Laboratory and maintained in SPF conditions. 8 to 12-wk-old male and female mice were used for experiments and were randomly allocated into treatment groups.

### Cell Lines.

Mouse NIH 3T3 cells (male), human A549 cells (male), and African green monkey Vero cells (female) were purchased from ATCC (#CRL-1658; #CCL-185; #CCL-81) and maintained in complete culture media: MEM supplemented with 10% heat inactivated fetal bovine serum (FBS, Sigma, US Origin), 2 mM L-glutamine, 100 μg/mL of penicillin, and 100 μg/mL of streptomycin. Human primary nTERT transformed keratinocytes were provided by Nir Drayman (UC Irvine), and maintained in keratinocyte serum-free media (ThermoFisher). Primary human dermal fibroblasts were obtained commercially from neonatal male donors (Cascade Biologics, #C0045C; ATCC, #CRL-2522). Cells were cultured in DMEM, supplemented with 10% FBS and 5% penicillin/streptomycin. All cells were kept at 37 °C and 5% CO_2_ in a humidified incubator, and passed every 3 to 4 d using 0.05% trypsin-EDTA. Cell cultures tested negative for *Mycoplasma*.

### Viruses.

HSV-1 strain KOS (wild type) and the mutants *d*109 (16), 8ΔGFP (17), and dl38 were provided by David Knipe (HMS), UL41 null and rescued strains (UL41NHB and UL41NHB-R) (18) were provided by David Leib (Dartmouth). Influenza A virus strain WSN 1933 was acquired from the BEI repository (#NR-3688). Viruses were propagated as previously described (58).

### Proteomics.

4.0 × 10^6^ cells were seeded in 100 × 20 mm Corning Falcon Tissue Culture Dishes. After 24 h, cells were infected with viruses at MOI 10. At the specified time points, cells were washed with PBS and lifted with TrypLE for 3 min. Cells were collected with 10 mL of PBS, spun at 200 g for 5 min, and washed 3× with 10 mL of ice-cold PBS. The cell pellet was resuspended in 200 µL of lysis buffer composed of 6 M Guanidine Hydrochloride (Chem-Impex), 2% Hexadecyltrimethylammonium Bromide (Millipore Sigma), 100 mM Na + EPPS pH 8.0 (Millipore Sigma), 10 mM TCEP HCl (pH = 7.5) (Oakwood Chemical), and 1 roche tablet cOmplete per every 12.5 mL of lysis buffer. Cell lysates were prepared, LC–MS/MS was performed, and analysis and search were done as we previously described, with some modifications (59) (*SI Appendix, Extended Methods*).

### RNA-Sequencing.

2.5 × 10^5^ mouse NIH 3T3 cells were seeded in 12 well VWR Cell Culture plates. The next day, cells were infected with viruses (MOI10). At the specified time points, cells were washed with PBS and lifted with TrypLE. As control, untreated (mock) samples were collected for each time point, as well as for time point 0 hpi. Cells were collected in complete cell culture medium. For each sample, cells were counted and 2.5 × 10^5^ cells were transferred into LoBind microcentrifuge tubes (Eppendorf), spun at 400 g for 4 min, and washed with PBS. Cell pellets (~10 µL) were resuspended with lysis buffer (Zymo DNA/RNA Shield; 55 µL). Finally, Invitrogen ERCC RNA Spike-in Mix (Mix1, final dilution: 7,500-fold) was added. Samples were submitted at room temperature for bulk RNA-sequencing to Plasmidsaurus. cDNA, library prep, read mapping, and normalization/analysis are described in detail in the Extended Methods.

### Gene Set Enrichment Analysis.

Preranked GSEA was performed on both the bulk RNA-seq and proteomics datasets using gseapy (v1.0.5). For each of the four strains (*d*109, 8ΔGFP, dl38, wild type), genes/proteins were ranked by log₂ fold-change versus mock at 24 hpi: for RNA, CPM-normalized log₂FC was used; for protein, log₂FC versus the matched control timepoint (TMM-normalized abundance, averaged across two biological replicates). Enrichment was assessed against the MSigDB mouse Hallmark gene sets (mh.all.v2026.1.Mm.symbols.gmt, release 2026.1) using 1,000 permutations, with gene set size restricted to 15 to 500 members (60). Pathways were considered significantly enriched if FDR-adjusted q-value < 0.05 and |NES| > 1.5.

### Antiviral Signature.

The HALLMARK_INTERFERON_ALPHA_RESPONSE gene set from MSigDB (mouse gene symbols, mh.all.v2026.1.Mm) was used to define the antiviral ISG list. An antiviral immune score was computed as the mean log₂FC of all detected ISGs relative to the condition’s own 6 hpi expression level ± SEM. As irrelevant comparator gene sets we used HALLMARK_APOPTOSIS and HALLMARK_KRAS_SIGNALING_UP from the same database.

### Mouse Corneal HSV-1 Infection.

Male and female C57Bl6/J mice aged 8 to 12 wk were infected with HSV-1 vp26-tdTomato (42) via corneal debridement. Animals were lightly anesthetized using isoflurane inhalation, and then one cornea was centrally debrided on the epithelium using a 0.5 mm rotating burr. 4 × 10^6^ PFU of virus in 4 μL of PBS was applied to the debrided cornea. The contralateral eye was left untreated. After 48 h, the animals were humanely euthanized, and the eyes were removed and incubated while tumbling for 1 h in 2 μM Puromycin (ThermoFisher) diluted in complete culture media prewarmed to 37 °C. After 1 h, eyes were fixed in ice-cold 1.6% paraformaldehyde for 1.5 h. Corneas were then dissected from the rest of the eye and stored in 90% methanol at −20 °C.

### Imaging Cell Death or Viral Spread.

2.5 × 10^4^ cells were seeded in 96 well glass bottom plate with high performance #1.5 cover glass (CellVis) precoated with 1 to 2.5 μg/cm^2^ fibronectin (Sigma) (58). Cells were labeled with 20 ng/mL Hoechst 33342 (Thermo Fisher) and death was tracked by incorporation of 1:10,000 Sytox Green (Thermo Fisher) (58).

### Immunofluorescence Staining.

Cells were fixed with 1.6% paraformaldehyde for 10 min on ice, then washed with PBS and permeabilized with ice-cold 90% methanol for at least 1 h (58). Fixed and permeabilized cells were washed with PBS, and blocked [PBS, 0.3% Triton X-100, and 5% normal human serum (Jackson ImmunoResearch)] for 1 h at room temperature. Human serum blocks non-specific binding of full IgG to HSV-1 infected cells. In other instances, blocking buffer consists of PBS, 0.3% Triton X-100, and 3% Bovine Serum Albumin (Sigma). Cells were stained with antibodies for 1 h at room temperature or overnight at 4 °C. Where, indicated, cells were then washed 3× with blocking buffer, then incubated with secondary antibodies for 1 h and counterstained with DAPI. Cells were washed 3× with blocking buffer and 2× with PBS before imaging.

Corneas were first washed in PBS, then blocked overnight [PBS, 0.3% Triton X-100, and 5% normal mouse serum (Jackson ImmunoResearch)] at room temperature, light protected. Corneas were then stained with antibodies and DAPI for 36 to 48 h in blocking buffer, tumbling at 4 °C, light-protected. Corneas were washed for 24 h with PBS before imaging.

### RNA FISH.

We designed custom oligonucleotide probe sets (IDT) complementary to our genes of interest using a custom MATLAB software (available at https://flintbox.com/public/project/50547/) (61). We pooled 15 to 32 oligonucleotides per gene, after which an amine group was added on the 3’ end with terminal deoxynucleotidyl transferase (TdT) and coupled to a fluorophore (Cy3 or Atto647N). Single-molecule RNA FISH was performed as described (62). Cells were fixed in 4% formaldehyde solution for 10 min and permeabilized with 70% ethanol for 1 h, at room temperature. We next washed the cells with 10% formamide and 2× SSC. For hybridization, we applied the RHA FISH probes in 10% formamide, 2× SSC, and 10% dextran sulfate overnight at 37 °C. Before imaging, the samples were washed twice and counterstained with DAPI.

### Puromycin Incorporation.

Cells were prepared with the indicated treatments, and then pulsed for 30 min with 2 μM Puromycin (ThermoFisher) before fixing and permeabilizing (34).

### Generation of CFLAR Overexpression Cells.

To generate a plasmid for lentivirus production of the Raji cFLAR isoform (murine cFLAR-R), we ordered a gene block with the sequence encoding this isoform (based on ref. 63) from IDT. The sequence was amplified (Q5 polymerase–NEB) and introduced in the NotI-XbaI sites of the parental vector (Addgene #90237) using Gibson Assembly (NEB).

Lentiviral transduction was used to generate NIH-3T3 cells stably overexpressing murine cFLAR^R^-T2A-H2B-iRFP^670^ or the control H2B-iRFP^670^. To produce lentivirus, HEK293T cells were transfected using optiMEM (Gibco) and Lipofectamine 3000 (Thermo Fisher Scientific) with the lentiviral plasmid of interest and packaging vectors psPax (Addgene #12260) and pMD2.G (Addgene #12259). The transfection medium was refreshed 1 d after transfection. The lentivirus-containing supernatant was collected 2 d after transfection. NIH-3T3 cells were seeded 1 d before transduction and grown to ~60% confluency. To transduce, the medium was replaced with lentiviral supernatant with Polybrene (10 μg/mL; Millipore) and the cells were spin-infected for 90 to 120 min at 2,000 rpm at 25 °C. After spin-infection, the cells were washed with PBS and cultured for 7 d in culture medium before further selection. Transduced cells were FACS-sorted based on iRFP^670^ expression.

### Antibodies.

anti-ICP4 (clone 10F1, Abcam), anti-IAV Nucleoprotein (clone AA5H, Bio Rad), anti-Puromycin-Alexa 488 or 647 (clone 2A4, BioLegend). All antibodies were used at 1:300 dilution of the stock. All polyclonal secondary antibodies were purchased from Jackson Immunoresearch and F(ab’)_2_ fragments were used at a concentration of 10 μg/mL. goat anti-Mouse IgG-Alexa 647 (cat: 115-606-003), goat anti-Mouse IgG-Alexa 594 (cat: 115-586-003).

### Microscopy.

Images were collected on a confocal Nikon Eclipse Ti2E-inverted microscope equipped with a Nikon CFI Plan Apo λ 20×, numerical aperture (NA) 0.75 objective lens. Images were acquired with a CrestOptics X-Light V3 light engine and a Photometrics Kinetix camera. For live-cell imaging, cells were maintained at 37 °C and 5% CO₂ in a humidified environmental chamber. All imaging was accomplished using custom automated software written using Python and Micro-Manager.

For RNA FISH imaging, samples were imaged on a Nikon Ti-E with a 60× Plan-Apo objective with 1.4 NA. Nikon Elements imaging software was used to stitch multitiled images.

### Treatment with Cytokines, Drugs, and Small Molecules.

Cells were treated with the following concentrations of molecules, unless otherwise indicated. Mouse TNFα (5 ng/mL, Peprotech), Human TNFα (5 ng/mL, Peprotech), cycloheximide (CHX) (20 μM, Sigma), Z-VAD(Ome)-FMK (30 μM, Cayman chemical), Necrostatin-1 (30 μM, Cayman chemical), Obatoclax (10 μM, Cayman chemical), A1210477 (10 μM, Cayman chemical), LCL-161 (5 μM, Cayman chemical), Thapsigargin (1 μM, Cayman chemical), Halofuginone (500 nM, Ralph Mazitschek, MGH), L-Proline (4 mM, Sigma).

### UV Irradiation.

Cells were irradiated in PBS for 15 s with a UV lamp set to 10 J/m^2^/s. After irradiation, complete media was replaced.

### Transfections and siRNA Knockdown.

For transfection of poly dA-dT (Invivogen) and HSV-1 *Vhs* (64), 2.5 × 10^4^ cells were seeded in 96 well glass bottom plate and adhered overnight. 200 ng/well of plasmids or poly dA-dT were transfected with Lipofectamine 3000.

For siRNA knockdowns, cells were sequentially knocked down. First, six well plates were seeded at a density of 1.25 × 10^5^ per well, then transfected with either a negative control (scramble) siRNA (Thermo Fisher #AM46111) or a pooled combination of three *Cflar*-targeting siRNAS (Thermo Fisher #’s 66164, 66073, and 65977) targeting exons 2, 3, and 5. siRNAS were all transfected at a final concentration of 10 nM using Lipofectamine 3000 in absence of P3000 reagent. After 24 h, cells were trypsinized and reseeded at 2.5 × 10^4^ cells per well in 96 well plates and simultaneously transfected again with the same final concentrations of siRNAs. Experiments were initiated 24 h after the second transfection.

### Image Analysis.

Automated image analysis was accomplished using custom software written in Python (58). Image analysis software is available from our GitHub repository: https://github.com/oylab/oyLabImaging. We segmented individual cell nuclei using a seeded watershed or Stardist (65) algorithm applied to the nuclear dye. Incorporation of a nucleic acid stain was used to call cell death based on an increase in fluorescence intensity. The threshold for death was determined by comparing to uninfected or untreated control conditions where cell death is minimal. We used the same strategy to call infected cells based on the fluorescence intensity of tagged HSV-1. To determine the fraction of infected or dead cells, we divided the number of dead cells at each timepoint by the total number of cells identified by segmentation at the indicated frame.

For RNA FISH, we used the NimbusImage platform (61), which is available here: https://github.com/arjunrajlaboratory/NimbusImage/ and hosted here: https://www.nimbusimage.com/. We used the Cellpose and Cellpose retrain tools (66–68) to detect cells and the Piscis and Piscis retrain tools (69) to detect mRNA spots. We connected each mRNA spot to the nearest cell using the “Connect to nearest tool” as described in the NimbusImage documentation.

### Mathematical Modeling.

We write a stochastic differential equation that incorporates TNFα triggered procaspase-8 cleavage, autocatalysis of procaspase-8 by caspase-8 dimers, inhibition of caspase-8 by free c-FLIP, natural caspase-8 degradation, and stochastic biochemical noise. TNFα signaling is modeled as a Hill function with coefficient 1 and EC_50_ of 1 ng/mL. Noise is modeled as a simple additive Gaussian process. We use a quasi-steady state approximation to couple the levels of free c-FLIP to the current levels of caspase-8. Please see the extended methods for model details and *SI Appendix*, Table S1 for model parameters.

To arrive at a population wide measurement of survival, we used the python package py-pde to solve the associated Fokker–Planck equation, which describes the time evolution of the probability density function of caspase-8 activity, and measured the fraction of live cells in the population as a function of time. The initial conditions were chosen to be the steady state distribution under conditions of maximal c-FLIP translation and no TNFα signaling. The fraction of dead cells was calculated as the total probability of having caspase-8 activity greater than 50% of its maximal value as a function of time. Death rates were extracted by fitting these curves to a simple exponential fit. The code to fully execute the model is available from our GitHub repository: https://github.com/oylab/TNFGuardModels.

### Quantification and Statistical Analysis.

All relevant data are shown as mean ± SD, SEM, or mean ± 95% CI of relevant fit. Statistical tests were selected based on appropriate assumptions with respect to data distributions and variance characteristics.

## Supplementary Material

Appendix 01 (PDF)

Dataset S01 (CSV)

Dataset S02 (CSV)

Dataset S03 (CSV)

Dataset S04 (CSV)

## Data Availability

Proteomics, RNA sequencing, and all software used in this paper including for image analysis and modeling data have been deposited in PRIDE, GEO, and Github (PXD064114, token: WFszDSsiqUC5 (70); PXD053765, token: RAlKaFtD3ZQA (71); http://proteomecentral.proteomexchange.org/ (72); https://github.com/oylab (73); https://github.com/oylab/oyLabImaging (74); GEO GSE339037 (75), token: ivedikcojhitdwh). All other data are included in the article and/or supporting information.
