# Supplementary material for: c-FLIP loss links arrest of gene expression to TNFα-dependent antiviral guard immunity
Source: Proc Natl Acad Sci U S A. 2026 Oct 22;123(43):e2600336123. doi: 10.1073/pnas.2600336123 (PMC13715744; doi:10.1073/pnas.2600336123)
Supplement: Supplementary file 1 [file pnas.2600336123.sapp.pdf]

Supporting Information for
c-FLIP Loss Links Arrest of Gene Expression to TNFα-Dependent Antiviral Guard Immunity

Matthew Sonnett*, Sanne Boersma*, Madeline E. Ward, Laura C. Van Eyndhoven, Edoardo Centofanti, Max Brambach, Changhong Zhou, David M. Knipe, Arjun Raj, Alon Oyler-Yaniv, and Jennifer Oyler-Yaniv

*equal contribution
Jennifer Oyler-Yaniv, Alon Oyler-Yaniv
Email: jen_oy@hms.harvard.edu; alon_oy@hms.harvard.edu

This PDF file includes:

- Supporting text
- Figures S1 to S3
- Table S1
- Legends for Datasets S1 to S4
- Supplemental References

Other supporting materials for this manuscript include the following:

- Datasets S1 to S4

Supporting Information Text
Extended Methods

RNA-seq sample prep and read mapping: After extraction of poly-adenylated mRNAs from 50µl per sample, cDNA is generated via reverse transcription and second-strand synthesis, followed by tagmentation, library indexing and amplification. Next, libraries were sequenced using Illumina NovaSeq (20M raw reads and 10M deduplicated reads per sample). To perform 3'-end counting, the following analyses were performed: 1) FastQ generation and demux using BSL Convert (v4.3.6) and fqtk (v0.3.1), 2) Read-filtering using FastP (v0.24.0), including poly-X tail trimming and 3' quality-based tail trimming (Phred quality score>15, and length>50 bp), 3) reference genome alignment using STAR aligner (v2.7), including non-canonical splice-junction removal. Mouse GRCm39_114 was used as a reference genome, supplemented with KOS-strain HSV-1 transcriptome (as defined in (1)), and supplemented with ERCC Spike-in Mix1 references, 4) BAM files coordinate sorting using samtools (v1.21), 5) UMI-based deduplication using UMICollapse (v1.1.0), 6) Mapping quality control using RustQC (v0.2.1), including QC reporting using MultiQC (v1.33), and 7) gene-expression quantification using featureCounts (v2.1.1) with strand-specific counting, multi-mapping read fractional assigning, exons and three prime UTR as the feature identifiers, and grouping by gene_id.

RNA-seq normalization approaches:

Normalization: Two normalization strategies were applied in parallel. For spike-in normalization, a per-sample size factor was computed as the ratio of that sample's total ERCC synthetic RNA counts to the geometric mean of ERCC totals across all samples; host gene counts were then divided by this size factor. This corrects for both sequencing depth and global changes in cellular RNA abundance caused by viral infection. For depth-only normalization, host gene counts were divided by the total host-mapped read count and multiplied by 10^6 to yield counts per million host reads (CPM), correcting for sequencing depth only.

Fold-change relative to mock: For each sample, $\log_2$ fold-change versus mock ($\log_2$FC) was computed as $\log_2$(normalized counts + 1) − $\log_2$(matched mock counts + 1), where mock was matched by timepoint and biological replicate. Per-replicate $\log_2$FCs were averaged across replicates. Genes were considered regulated if $|\log_2 FC| > 1$ (> 2-fold) in at least one timepoint.

LC-MS Sample Prep: Samples were sonicated for 10 rounds of 15s at an amplitude of 60 on ice and clarified at 20k g for 15min at 4°C. Disulfide bonds were reduced by addition of 5 mM Dithiothreitol (DTT) (Oakwood Chemical) at 60°C for 15min. 50mM of iodoacetamide (Millipore Sigma) was added to alkylate cysteines and samples were kept in the dark for 1h, then quenched by addition of 30mM DTT. A chloroform/methanol protein precipitation was done as described (2). The protein disc was re-suspended at ~2µg/µL in 6M GuHCl / 10mM EPPS pH 8.5. 50µg of protein from each sample was diluted to 2M GuHCl by addition of 10 mM EPPS pH 8.5, and digested for 14h with 20ng/µL LysC (Wako). Samples were diluted to 0.5 M GuHCl and 20ng/µL LysC with 10ng/µL Trypsin (Sequencing grade, Promega) and incubated at 37°C for 16h. Solvent was removed in vacuo and each sample was re-suspended in 500mM EPPS pH 8.0 at ~1µg/µL. 20µL of TMTPro (Thermo) (20µg/µL in anhydrous Acetonitrile (ACN)) was added to 50µg of peptides and incubated at room temperature for 2h. 10µL of 5% hydroxylamine (Millipore Sigma) was added to quench the reaction. Samples were combined and the solvent removed in vacuo. Samples were acidified to pH ≤ 1 with TFA, clarified at 20k g at 4°C for 10min and desalted using a seppak (Waters). The seppak was wet with 1mL of methanol, followed by 1mL of 1% Formic acid (FA), then washed twice with 1mL of 1% FA and sample eluted into a 2 mL Eppendorf with 1mL of 35% ACN/1% formic acid. Solvent from the eluent was removed in vacuo, the sample was re-suspended in 140µL of 10mM Ammonium Bicarbonate pH 8.0, and clarified in an ultracentrifuge at 70,000 rpm for 30 minutes at 4°C. 100 µL of supernatant was fractionated by

reverse phase chromatography as previously described (3) into a 96-well plate and 24 fractions were created, desalted by stage-tips (4) and ~10μg of peptides were subjected to LC-MS/MS.

LC-MS/MS: A Thermo Orbitrap Eclipse was operated in data-dependent mode with a survey scan of 300-1100 m/z at an Orbitrap resolution of 120,000 with a 200 ms max injection time, AGC target of 1e6, and a Lens RF of 60%. The Eclipse was connected to a Thermo EASY-nLC 1200 HPLC. Individual runs were 4.5 hours in length with a gradient of 3% to 18% B (Solvent A 0.125% FA, 2% DMSO and Solvent B 0.125% FA, 80% ACN, 2% DMSO). Chromatography was performed with in house packed 100-360 μm inner-outer diameter microcapillary columns, which were manually packed with ~0.5 – 1cm of magic C4 resin followed by 40cm of 1.9cm Dr. Maisch GmbH C18-AQ resin (Part # r119.aq.0001)(5). An in-house machined column heater was used and kept at 60°C. Peptides were ionized with a 2.6 kV voltage applied to a PEEK microtee. Each MS/MS run was composed of four different experiments with different FAIMS (6) CVs (-32, -42, -52, -62). FAIMS was operated at standard resolution with a total carrier gas flow of 3.5 L/min. A dynamic exclusion window of 60 seconds with a filter of +/- 10 PPM was used. MS/MS isolations were performed with a quadrupole isolation window of 0.4 m/z, and peptides were fragmented with an HCD collision energy of 37%. The MS/MS scan was acquired with an orbitrap resolution of 50,000 with a first mass of 110 m/z. An AGC target of 50,000 was used with a maximum injection time of 120 ms. The low m/z TMT-Pro reporter ions were quantified with a 20 ppm mass tolerance and corrected for isotopic impurities as described previously (7). Experiment shown in 1E-F and S1B was done as above except the MS/MS scan was acquired with an Orbitrap resolution of 15,000 and an additional MS3 scan was added with an MS isolation window of 0.7 m/z, Orbitrap resolution of 60,000 and 5 SPS precursors were analyzed.

***LC-MS/MS analysis*:** The comet search engine was used instead of Sequest. A precursor peptide mass tolerance of 50 ppm was used allowing for the variable modifications of oxidation of methionine (+15.994914 Da) and deamidation of asparagine (+ 0.984016 Da). Iodoacetamide on cysteine (+ 57.02146372 Da) and TMT-Pro (+304.20660 Da) on n-termini and lysines were set as static modifications. The target-decoy approach was used to set the peptide false discovery at 1% (8) and these peptides were further filtered to obtain a 1% FDR on the protein level using protein pickr (9). A minimum Sum Sn of 400 for all conditions was required for each protein to be considered quantified. The sum of all TMT-reporter ions of each protein for each condition were normalized to be equal. The TMT reporter Sn for each protein row was normalized to sum to one. Peptides with an MS1 isolation specificity of >= 0.95 were used preferentially for protein quantitation. Contaminants and target-decoy matches were removed.

Mathematical modeling details.

<u>Single cell stochastic simulations of caspase-8 dynamics:</u>
Single-cell caspase-8 dynamics were simulated by numerically integrating the Langevin equation using the Itô-Euler scheme implemented in the sdeint Python package (sdeint.itoEuler). Simulations were run over 48 hours with 100,000 equally spaced time points, giving a time step of $\Delta t = 4.8\times10^{-4}$ h. cFLIP inhibition was treated in quasi-steady-state (QSS), such that the effective inhibitory term depends on the ratio of the cFLIP translation rate to the sum of the cFLIP inhibition constant and current caspase-8 level. The drift term includes caspase-8 self-activation, saturating TNF-driven activation, saturating cFLIP-mediated inhibition, and linear degradation, with a logistic regularization factor (1 - x/100) suppressing unphysical growth at large caspase-8 values. Additive Gaussian white noise with amplitude σ = 2 nM $h^{-1/2}$ was used to represent stochastic fluctuations in signaling. All simulations were initialized at x_0 = 0 nM. Ten independent trajectories

were simulated for each combination of TNF concentration and cFLIP translation rate, and trajectories that crossed below -2 nM were discarded and rerun to avoid unphysical states.

Itô-Euler scheme:

The SDE integrated at each time step Δt is:

$$x(t + \Delta t) = x(t) + f(x, t) \cdot \Delta t + \sigma \cdot \Delta W$$

where $\Delta W \sim N(0, \Delta t)$ is a Wiener increment, and the drift term $f(x, t)$ is:

$$f(x, t) = (\alpha x^2 - \frac{k_{flip} \cdot x}{\beta_{flip} + x} + \frac{k_T T}{1+T} - \beta x)(1 - \frac{x}{100})$$

Fokker-Planck simulations of caspase-8 probability distributions:

The Fokker-Planck equation was solved numerically using the py-pde Python package, on a 1D grid spanning -1 to 120 nM with 5000 equally spaced points, using an explicit Euler scheme with adaptive time-stepping. The drift term was precomputed on the grid and includes a logistic regularization factor (1 - x/100) to suppress unphysical probability flux at large caspase-8 values. Boundary conditions were set to zero-value at the lower boundary and zero-derivative (Neumann) at the upper boundary for the drift term, and zero-derivative (Neumann) at both boundaries for the diffusion term. The initial condition for all parameter combinations was the steady-state probability distribution obtained by equilibrating the system at maximum cFLIP (b = 5 nM h^{-1}) and zero TNF, starting from a narrow Gaussian centered at x = 0. Solutions were then computed for all combinations of 11 cFLIP translation rates (linearly spaced, 0–5 nM h^{-1}) and 12 TNF concentrations (logarithmically spaced, 0–100 ng/ml) over 24 hours. Death was defined as the probability mass above a caspase-8 threshold of 50 nM, and per-condition death rates were extracted by fitting the log-survival curve to a linear function using scipy.stats.linregress.

Fokker-Plank Equation:

$$\frac{\partial P(x,t)}{\partial t} = \frac{\sigma^2}{2} \frac{\partial^2 P(x,t)}{\partial x^2} - \frac{\partial}{\partial x}[f(x, t) \cdot P(x, t)]$$

Parameter justifications

This model is a reduced, single-variable stochastic description of active caspase-8 dynamics, and several parameters should be interpreted as effective, rather than direct biochemical rate constants. The quasi-steady-state treatment of cFLIP is supported by its rapid turnover, with reported half-lives on the order of tens of minutes to a few hours, justifying its representation as an effective inhibitory capacity rather than an explicitly modeled species. The TNF half-saturation parameter (K_dT = 1 ng/mL) is likewise an effective cellular EC50 for pro-death signaling rather than a ligand–receptor binding affinity, consistent with the observation that low-ng/mL TNF is sufficient to induce measurable priming while spanning the experimentally relevant dynamic range (cite our nature comms paper). The caspase-8 self-activation parameter (α) captures the well-established autocatalytic and cooperative nature of caspase-8 activation and was chosen such that the balance between amplification and linear loss (β = 0.5 h^{-1}) occurs near ~10 nM caspase-8, producing a switch-like regime in which TNF alone primes but does not deterministically drive commitment. Consistent with this, the TNF-driven activation rate (k_T) was selected so that, in the absence of amplification, TNF shifts the system into a sensitized but sublethal state (steady state ~10 nM), enabling stochastic or feedback-driven transitions to high activity. The cFLIP inhibition constant (a) sets the scale over which inhibition is relieved and is chosen in the low-nM range to reflect strong suppression at low caspase-8 levels with escape upon amplification, while the cFLIP production term (b) is varied across a range comparable to k_T to span regimes from protection to sensitization. The noise amplitude (σ) captures cell-to-cell

variability and temporal fluctuations, chosen to generate heterogeneous activation dynamics without overwhelming the deterministic structure of the system.

Supplementary Figure 1

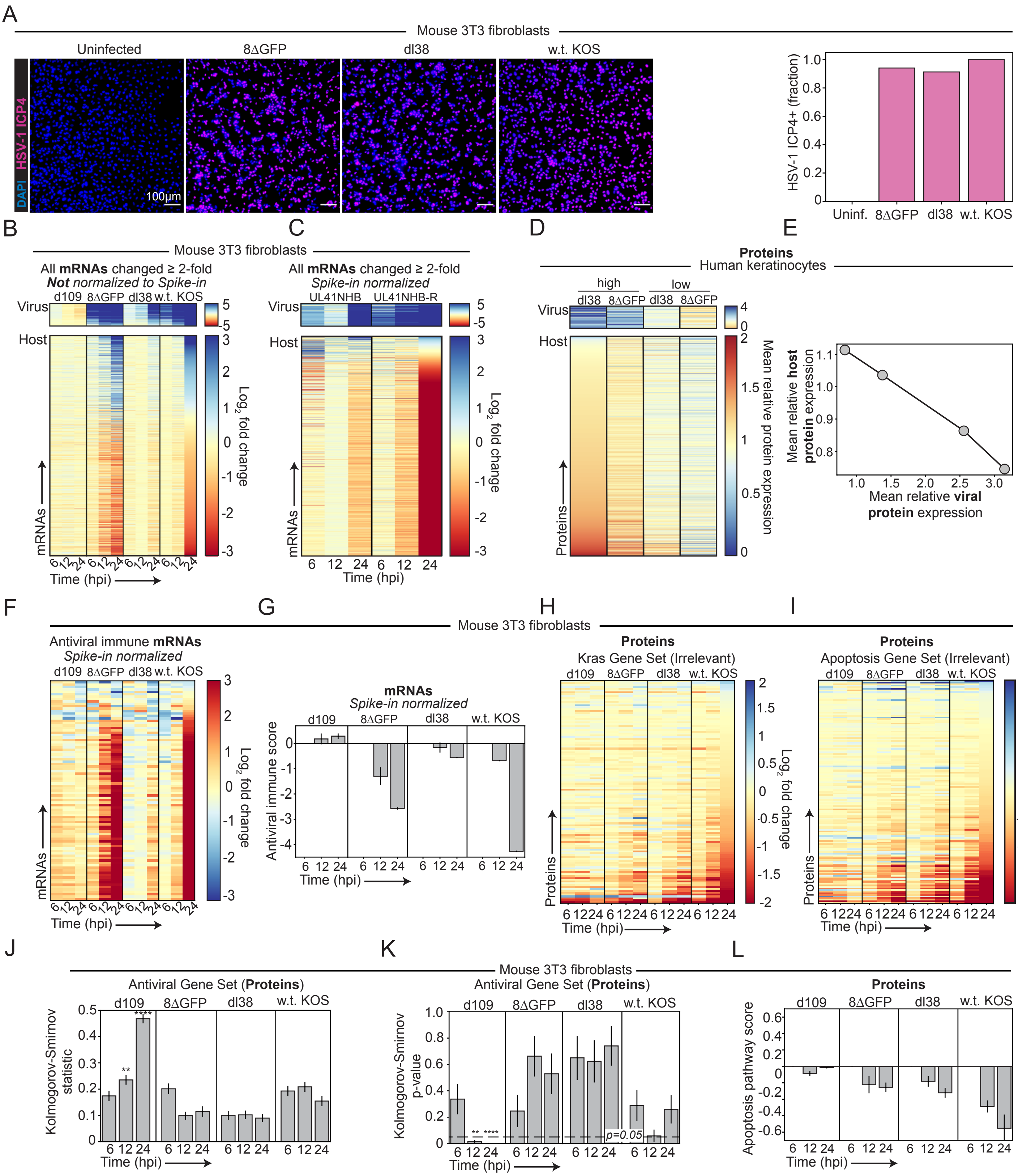

Fig. S1. (A) Representative images and quantification of HSV-1 infected mouse 3T3 fibroblasts (based on ICP4 immunofluorescence) after overnight infection with MOI 10 of each of the different viral mutants. **(B)** Heatmap of differentially expressed genes (|log2fc|>2 compared to matching mock infection) in CPM-normalized mRNA sequencing data of mouse 3T3 fibroblasts infected with the indicated HSV-1 strains. **(C)** Heatmap of differentially expressed genes (|log2fc|>2 compared to matching mock infection) in spike-in normalized mRNAseq data of mouse 3T3 fibroblasts infected with the indicated HSV-1 strains (UL41NHB or UL41NHB, vhs null or rescued, respectively). **(D)** Heatmap showing protein expression for human keratinocytes infected with high or low levels of the dl38 or 8ΔGFP strains. **(E)** Quantification of mean viral protein versus mean host protein from (D). **(F)** Heatmap of antiviral genes in spike-in normalized mRNAseq data of mouse 3T3 fibroblasts infected with MOI 10 of the indicated HSV-1 strains **(G)** Quantification of the antiviral immune score for mouse 3T3 fibroblasts infected with the indicated HSV-1 strains. To compute the score, we took the mean expression of all mRNAs on our antiviral immune list ± s.e.m. **(H-I)** Heatmaps showing protein expression for an irrelevant set of genes associated with KRAS signaling (H) or apoptosis signaling (I) for infected mouse 3T3 fibroblasts. **(J)** Quantification of the Kolomgorov-Smirnov statistic for all proteins in our antiviral immune signature compared to 10,000 randomly-selected genes for mouse 3T3 fibroblasts. ** $p \leq 0.01$, **** $p \leq 0.0001$. Data show the mean ± s.d. of 100 random samplings. **(K)** Quantification of the Kolomgorov-Smirnov p-value for the same conditions as in E. Data show the mean ± s.d. of 100 random samplings. **(L)** Quantification of the apoptosis protein expression score for mouse fibroblasts infected with each of the viral mutants. To compute the score, we took the mean expression of all proteins annotated in the MSigDB Hallmark apoptosis pathway list ± s.e.m. for each protein.

Supplementary Figure 2

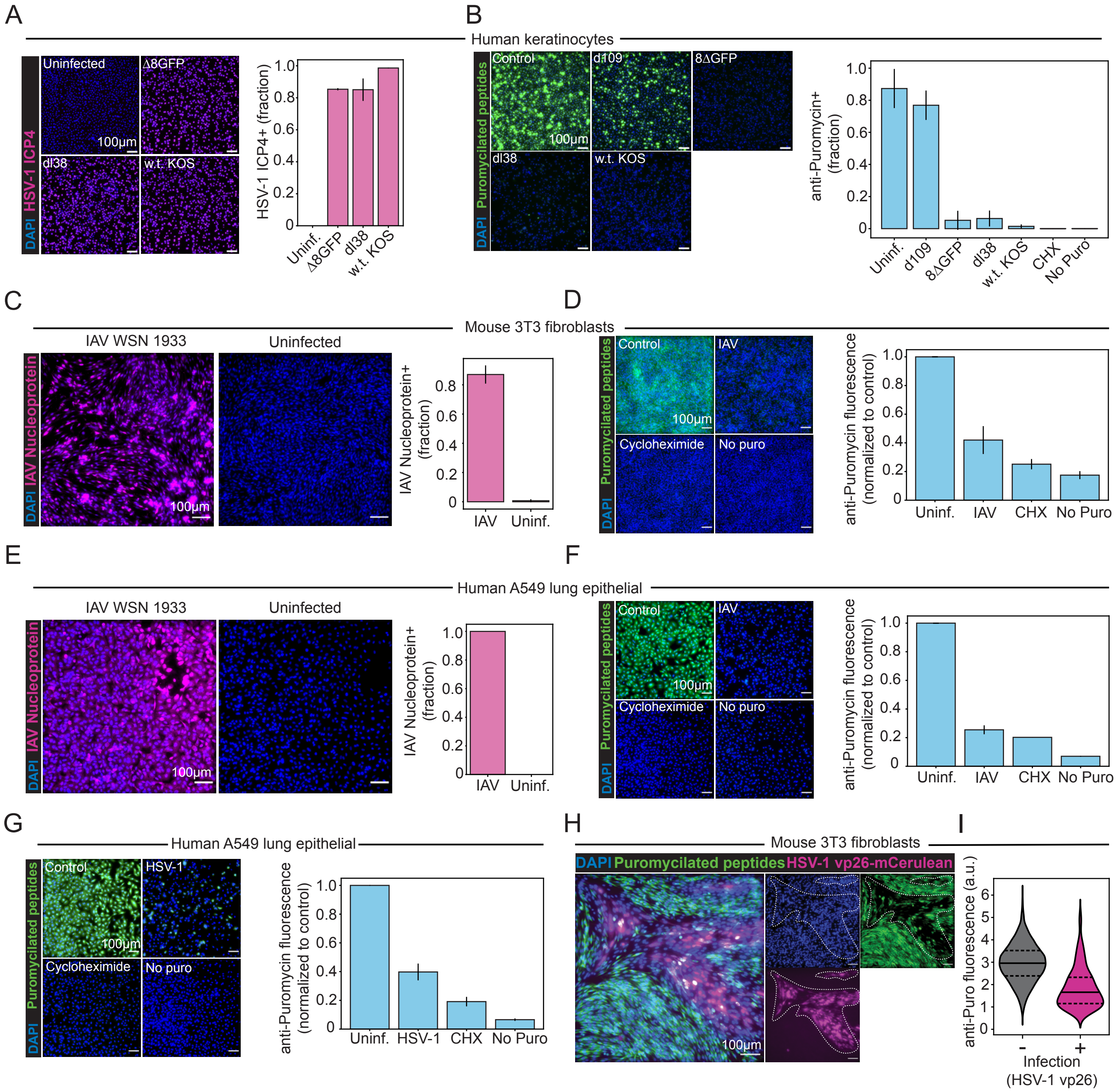

Fig. S2. (A) Representative images and quantification of HSV-1 infected cells (based on ICP4 immunofluorescence) after overnight infection of human keratinocytes with MOI 10 of each of the different viral mutants (KOS). **(B)** Representative images and quantification of anti-puromycin staining for human keratinocytes infected overnight with MOI 10 of each of the indicated HSV-1 viral mutant strains. **(C)** Representative images and quantification of IAV infection (strain WSN/1933) based on Nucleoprotein (NP) immunofluorescence after overnight infection of 3T3 fibroblasts with MOI 10. **(D)** Representative images and quantification of anti-puromycin staining for 3T3 fibroblasts infected overnight with MOI 10 of IAV. **(E)** Representative images and quantification of IAV infection (based on NP immunofluorescence) after overnight infection of A549 cells with MOI 10 strain WSN/1933. **(F)** Representative images and quantification of anti-puromycin staining for A549 cells infected overnight with MOI 10 of IAV strain WSN/1933. **(G)** Representative images and quantification of anti-puromycin staining for A549 cells infected overnight with MOI 10 of HSV-1 w.t. strain KOS. **(H)** Representative images of anti-puromycin staining for cells infected overnight with MOI 3 of HSV-1 vp26-mCerulean (10) (KOS). **(I)** Violin plot of anti-puromycin fluorescence for VP26-mCerulean+ (infected) or - (uninfected) cells. The viral plaque edge is marked with a dotted white line. Inner lines show the median ± quartiles of the data. Unless otherwise stated, for all of Fig S2, the data show the mean ± s.d. of multiple biological replicates.

Supplementary Figure 3

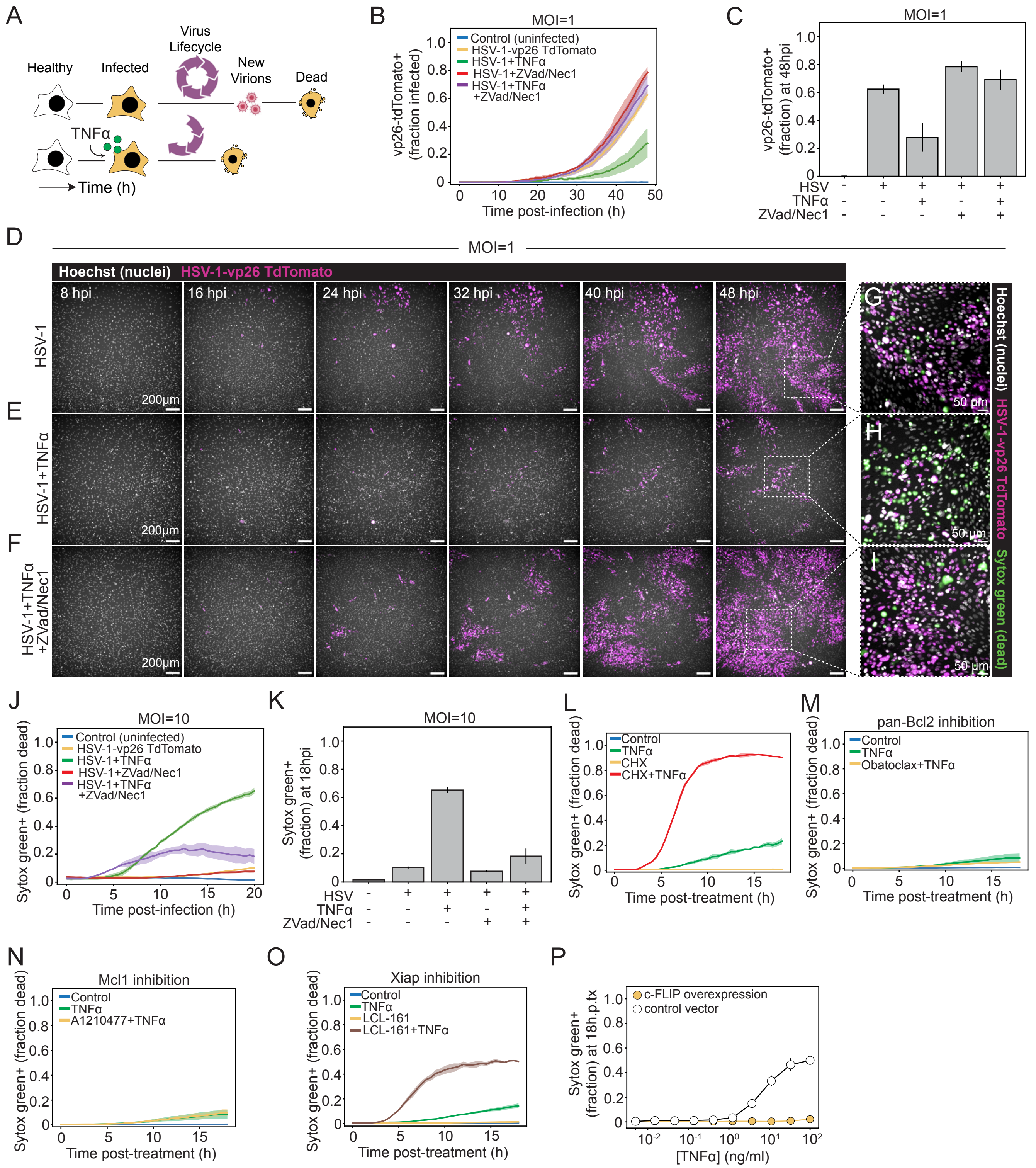

Fig. S3. (A) Cartoon diagram showing the principle. TNFα accelerates cell death after viral infection, which prevents completion of the viral life cycle and production of new infectious virions (11). **(B)** Quantification of infected cells over time (based on expression of HSV-1 VP26-tdTomato) after infection with MOI 1 for the indicated conditions. **(C)** Quantification of infected cells for each of the indicated conditions at 48hpi. **(D-F)** Representative images showing viral spread for 3T3 cells left untreated (D), treated with 5 ng/ml TNFα (E), or treated with TNFα and 30 μM each of Z Vad/Nec1 (F). **(G-I)** Close-up images showing cell death from indicated insets of larger images for each condition. **(J)** Quantification of cell death over time for 3T3 cells infected with MOI 10 of w.t. HSV-1 alone (KOS background), or HSV-1 with 5 ng/ml TNFα, HSV-1 with 30 μM each of ZVad and Nec1, or HSV-1,TNFα and ZVad/Nec1. **(K)** Quantification of dead cells for each of the indicated conditions at 18hpi. **(L)** Quantification of cell death over time for 3T3 cells treated with either 20 μM CHX or 5 ng/ml TNFα, or CHX with TNFα. **(M)** Quantification of cell death over time for 3T3 cells treated with either 5 ng/ml TNFα alone or TNFα combined with 10 μM of the pan-Bcl2 inhibitor Obatoclax. **(N)** Quantification of cell death over time for 3T3 cells treated with either 5 ng/ml TNFα alone or TNFα combined with 10 μM of the Mcl1 inhibitor A1210477. **(O)** Quantification of cell death over time for 3T3 cells either 5 ng/ml TNFα alone or TNFα combined with 5 μM of the Xiap inhibitor LCL-161. **(P)** Quantification of dead cells for control vector or c-FLIP overexpressing 3T3 cells at 18hp-tx with a dose titration of TNFα. For all of fig S3, plots show the mean ± s.d. of multiple biological replicates.

Tables

Table S1. Model Parameters

**Parameter**	**Symbol**	**Value**	**Units**	**Description**
Self-activation rate	α	0.05	nM^{-1} h^{-1}	caspase-8 positive feedback strength
cFLIP translation rate	b	0–5	nM h^{-1}	Rate of cFLIP production
cFLIP inhibition constant	a	1	nM	Half-saturation of cFLIP inhibition
caspase-8 degradation rate	β	0.5	h^{-1}	Linear degradation of caspase-8
TNF response amplitude	k_T	5	nM h^{-1}	Max TNF-driven activation rate
TNF EC50	K_dT	1	ng ml^{-1}	TNFR half-saturation concentration
TNF concentration	T	0–100	ng ml^{-1}	Extracellular TNF
Noise amplitude	σ	2	nM $h^{-½}$	Additive white noise strength

Dataset S1 (separate file). mRNA expression for mouse NIH-3T3 cells infected with HSV-1 strains. Cells were infected with MOI 10 of the indicated HSV-1 mutant strains and harvested at 6, 12, and 24hpi for mRNA sequencing.

Dataset S2 (separate file). Protein expression for mouse NIH-3T3 cells infected with HSV-1 strains - Replicate 1. Cells were infected with MOI 10 of the indicated HSV-1 mutant strains and harvested at 6, 12, and 24hpi for proteomics.

Dataset S3 (separate file). Protein expression for mouse NIH-3T3 cells infected with HSV-1 strains - Replicate 2. Cells were infected with MOI 10 of the indicated HSV-1 mutant strains and harvested at 6, 12, and 24hpi for proteomics.

Dataset S4 (separate file). Protein expression for n-TERT transformed keratinocytes infected with different doses of non-spreading HSV-1 strains. Cells were infected with high and low doses of HSV-1 strains 8ΔGFP and dl38, and were harvested at 24hpi for proteomics.

SI References

1. M. L. Szpara, *et al.*, Evolution and diversity in human herpes simplex virus genomes. *J. Virol.* **88**, 1209–1227 (2014).

2. D. Wessel, U. I. Flügge, A method for the quantitative recovery of protein in dilute solution in the presence of detergents and lipids. *Anal. Biochem.* **138**, 141–143 (1984).

3. A. Edwards, W. Haas, Multiplexed Quantitative Proteomics for High-Throughput Comprehensive Proteome Comparisons of Human Cell Lines. *Methods Mol. Biol.* **1394**, 1–13 (2016).

4. J. Rappsilber, M. Mann, Y. Ishihama, Protocol for micro-purification, enrichment, pre-fractionation and storage of peptides for proteomics using StageTips. *Nat. Protoc.* **2**, 1896–1906 (2007).

5. A.-D. Brunner, *et al.*, Ultra-high sensitivity mass spectrometry quantifies single-cell proteome changes upon perturbation. *Mol. Syst. Biol.* **18**, e10798 (2022).

6. D. K. Schweppe, *et al.*, Characterization and optimization of multiplexed quantitative analyses using high-Field Asymmetric-waveform ion mobility mass spectrometry. *Anal. Chem.* **91**, 4010–4016 (2019).

7. M. Sonnett, E. Yeung, M. Wühr, Accurate, Sensitive, and Precise Multiplexed Proteomics Using the Complement Reporter Ion Cluster. *Anal. Chem.* **90**, 5032–5039 (2018).

8. J. E. Elias, S. P. Gygi, Target-decoy search strategy for increased confidence in large-scale protein identifications by mass spectrometry. *Nat. Methods* **4**, 207–214 (2007).

9. M. M. Savitski, M. Wilhelm, H. Hahne, B. Kuster, M. Bantscheff, A Scalable Approach for Protein False Discovery Rate Estimation in Large Proteomic Data Sets. *Mol. Cell. Proteomics* **14**, 2394–2404 (2015).

10. L. Etienne, *et al.*, Visualization of herpes simplex virus type 1 virions using fluorescent colors. *J. Virol. Methods* **241**, 46–51 (2017).

11. J. Oyler-Yaniv, A. Oyler-Yaniv, E. Maltz, R. Wollman, TNF controls a speed-accuracy tradeoff in the cell death decision to restrict viral spread. *Nat. Commun.* **12**, 2992 (2021).
